# Aspects of Growth Hormone and Insulin-Like Growth Factor-I Related to Neuroprotection, Regeneration, and Functional Plasticity in the Adult Brain

**DOI:** 10.1100/tsw.2006.22

**Published:** 2006-01-18

**Authors:** N. David Åberg, Katarina Gustafson Brywe, Jörgen Isgaard

**Affiliations:** ^1^ Research Center for Endocrinology and Metabolism, Department of Internal Medicine, Sweden; ^2^ Department of Obstetrics and Gynecology, Perinatal Center, Sahlgrenska Academy, University of Göteborg, Göteborg, Sweden

**Keywords:** growth hormone (GH), insulin-like growth factor (IGF), brain, CNS, stem cells, neurogenesis, cell genesis, adult, neuroprotection, regeneration, plasticity, neurotransmitters, cerebral blood flow, cognition, memory, aging, exercise, stress, dendrite, angiogenesis, gap junctional communication, glucose metabolism, brain repair, transduction pathways

## Abstract

Apart from regulating somatic growth and metabolic processes, accumulating evidence suggests that the growth hormone (GH)/insulin-like growth factor-I (IGF-I) axis is involved in the regulation of brain growth, development, and myelination. In addition, both GH and IGF-I affect cognition and biochemistry in the adult brain. Some of the effects of GH are attributable to circulating IGF-I, while others may be due to IGF-I produced locally within the brain. Some of the shared effects in common to GH and IGF-I may also be explained by cross-talk between the GH and IGF-I transduction pathways, as indicated by recent data from other cell systems. Otherwise, it also seems that GH may act directly without involving IGF-I (either circulating or locally). Plasticity in the central nervous system (CNS) may be viewed as changes in the functional interplay between the major cell types, neurons, astrocytes, and oligodendrocytes. GH and IGF-I affect all three of these cell types in several ways. Apart from the neuroprotective effects of GH and IGF-I posited in different experimental models of CNS injury, IGF-I has been found to increase progenitor cell proliferation and new neurons, oligodendrocytes, and blood vessels in the dentate gyrus of the hippocampus. It appears that the MAPK signaling pathway is required for IGF-I—stimulated proliferation *in vitro*, whereas the PI3K/Akt or MAPK/Erk signaling pathway appears to mediate antiapoptotic effects. The increase of IGF-I on endothelial cell phenotype may explain the increase in cerebral arteriole density observed after GH treatment. The functional role of GH and IGF-I in the adult brain will be reviewed with reference to neurotransmitters, glucose metabolism, cerebral blood flow, gap junctional communication, dendritic arborization, exercise, enriched environment, depression, learning, memory, and aging.Briefly, these findings suggest that IGF-I functions as a putative regenerative agent in the adult CNS. Hitherto less studied regarding in these aspects, GH may have similar effects, especially as it is the main regulator of IGF-I *in vivo*. Some of the positive cognitive features of GH treatment are likely attributable to the mechanisms reviewed here.

## THE PRESENCE OF THE GH-IGF-I SYSTEM IN THE BRAIN

### CNS Expression of Receptors for GH and IGF-I

Several studies have addressed the expression and distribution of GH receptors (GH-R) and IGF-I receptors (IGF-I-R) in the brain. The first characterization was performed with ligand binding experiments and subsequently with mRNA and protein expression patterns, both using brain region homogenates and immunohistochemistry. Generally, the different methods show a comparatively similar distribution of receptor expression. GH-R immunoreactivity has been reported to be present in neurons, astrocytes, and oligodendrocytes[58] (Table 1, Fig. 1), and it appears that there is a partial agreement with expression for regions that abundantly coexpress IGF-I and IGF-I-R[42]. Particularly high expression of GH-R has been observed in the choroid plexus, hippocampus, and the hypothalamus in both rodents[58] and humans[37]. IGF-I-R is expressed in neuronal stem cells[171], but also appears to be present mostly in neurons, but also in glial cells throughout the brain, with the highest density in cerebral cortex and the striatum[32,162]. Like GH-R, the IGF-I-R is highly abundant in the choroid plexus as shown in both ligand binding experiments[25] and from IGF-I-R mRNA studies[51].

### Local Expression of GH and IGF-I in the Brain

Although the pituitary is considered to be the exclusive source for GH in the blood and liver-derived IGF-I constitutes the major part of circulating IGF-I, studies have suggested a local production of both GH and IGF-I in various parts of the brain.

In 1980, GH-like material was found outside the pituitary in hypophysectomized rats and primates[10,13] whose pituitary GH should have had time to be “washed out” (see also Table 1). The levels were about $10^{-6}$ less than in the anterior pituitary[13]. Using *in situ* hybridization, the presence of GH mRNA has been reported in the caudate putamen, the striatum, the ventral thalamus, the formation reticularis, the outer part of hippocampus, and the basal cortex[20]. Although controversial for a number of years, additional data during the last years have added support for at least a low-level expression of nonpituitary GH within various parts of the brain[74,219]. This finding is intriguing, although the biological significance of this local production of GH outside the pituitary remains to be investigated.

IGF-I immunoreactivity is widespread in all types of neuronal cells in the brain[72]. Findings that IGF-I expression in the CNS is particularly high during fetal development and reaches its peak expression during the first two postnatal weeks, predominantly in neurons, but also in glial progenitors, suggest that this growth factor may play a role in the development of the nervous system[27,31,35,41]. Moreover, IGF-I expression in the brain is partly transient corresponding to regions and periods of axon outgrowth, dendritic maturation, and synaptogenesis[42].

In conclusion, there are multiple possibilities for direct actions of peripherally derived or locally produced GH, as well as endocrine or autocrine/paracrine effects of IGF-I on the CNS. However, at present, the relative importance of these different pathways of GH and IGF-I action remains unclear.

### IGF Binding Proteins

The majority of circulating IGF-I is bound to a variety of different IGF binding proteins (IGFBPs), of which six have been characterized to date. After extensive research, a wide range of different functions for the IGFBPs have been proposed and they are considered to be of importance for the pharmacokinetics of IGF-I, both in circulation and at the tissue level. At the cell surface or in the extracellular cell matrix, IGFBPs can either inhibit or enhance the presentation of IGF-I to its receptor. Recently, reports have suggested additional IGF-I independent actions of the IGFBPs (for review, see [168]). Colocalization of IGFBPs and IGF-I has been reported to occur during brain development and has been suggested to be a mechanism for modulating the actions of IGF-I[119,131]. IGFBP-2[95], IGFBP-4[64], and IGFBP-5[64] appear to be the predominant binding proteins, although low expression of IGFBP-1[131], IGFBP-3[95],

**TABLE 1**
**Presence of GH, GH-R, IGF-I, IGF-I-R, GH Binding Proteins, and IGF Binding Proteins in Four Brain Regions***

| | ***Hypothalamus*** | | ***Cortex*** | | ***Hippocampus*** | | ***Cerebellum/Brain Stem*** | | ***Ref.*** |
|---|---|---|---|---|---|---|---|---|---|
| GH mRNA | ++ | Neuron | ++ | NE | + | NE | + | NE | [39], [20] |
| GH protein | ++ | NE | + | NE | + | NE | NE | | [13] |
| GHrec mRNA (adult) | ++ | Neuron (PeN, PVL) | –<br>+ | Neurons (frontal cortex)<br>Unidentified cells | ++ | NE | NE | | [43] |
| GHrec protein | ++<br>++ | Neuron<br>Ventr. Astr. | ++<br>–<br>++<br>++<br>++ | Neuron (2, 3, 5, 6)<br>Astrocyte (gen.)<br>Astrocyte (cing.)<br>Oligodendr (cing.)<br>Choroid plexus | ++<br>++ | Neuron<br>Ventr. Astr. | ++<br>+ | Neuron (Purkinje)<br>Neuron (gen.) | [58], postnatal day 10 |
| GHrec (ligand) | + | NE | +++<br>+ | Choroid plexus<br>Frontal cortex | ++ | NE | + | NE | [37], human data |
| IGF-I mRNA | + | NE | ++ | Neurons | ++ | Neurons (CA1-CA2, DG) | ++ | Neurons | [34], [42], [32] |
| IGF-I protein | ++<br>++ | Astrocytes<br>Tancytes | + | NE | NE | | NE | | [61], [80] |
| IGF-I rec. mRNA | ++<br>+ | Neuron (suprachiasmatic nucl.)<br>Astrocyte | ++<br>++<br>++ | Neuron<br>Choroid plexus<br>Vascular sheath | +++ | Cell type NE, but probably neurons (CA2-4, DG, GCL, Hilus | + | NE | [85], [51], [42], [32] |
| IGF-I rec protein | NE | | +<br>+++ | Neurons (2,3,5,6)<br>Ependymal cells | ++<br>+/-<br>+++ | Neurons (CA1-3)<br>Other cells ?<br>*In vitro*: adult hippocampal progenitor cells | NE | | [162], [171] |
| IGF-I rec. (ligand) | ++ | NE | ++<br>+++ | Layer 1,2<br>Choroid plexus | ++<br>+ | CA1, CA3, GCL<br>Hilus etc. | ++ | NE | [38], [25], [24] |
| GHBP | All cell types (neuronal, glial, ependymal) in the brain show GHBP IR, no detailed data supplied | | | | | | | | [47] |
| IGFBP (1-6) | Complex regulation and interaction with IGFs, synthesized by both neurons and astrocytes | | | | | | | | [104] |

*The table provides examples of GH and IGF-I effects and is not exhaustive, although many of the important references are included. Unless otherwise specified, the data refer to adult rodents.

+++, Intense; ++, moderate to intense; +, identifiable, but not intense; –, not identifiable.

Abbreviations: IR, immunoreactivity; NE, not examined, unknown, or not shown; Ventr. Astr., ventricular astrocytes (including ependymocytes); L(1-6), cerebral layer 1-6 as specified; gen., general; cing., cingulum; CA, cornu ammonis (in the hippocampus); DG, dentate gyrus (in the hippocampus); GCL, granule cell layer.

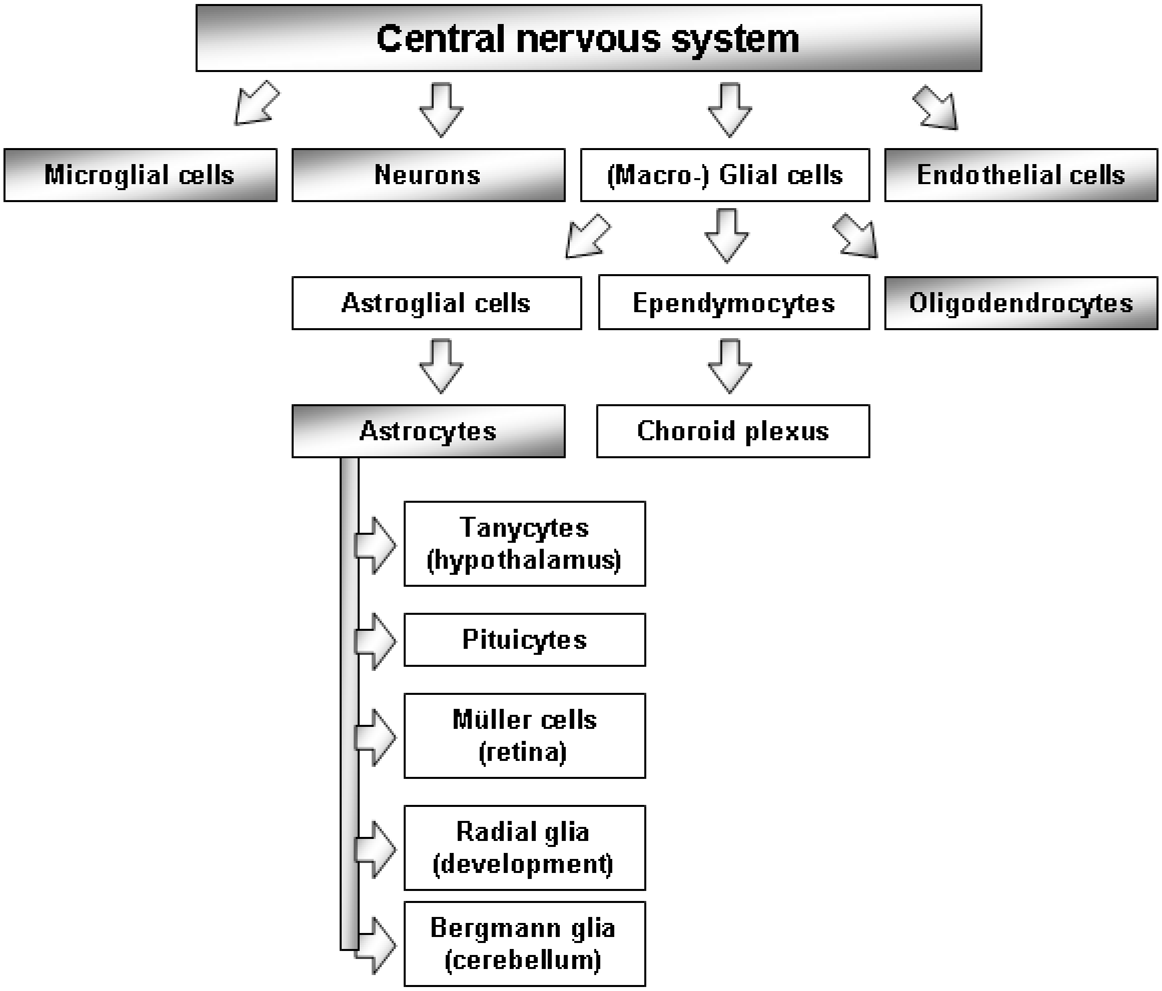

**FIGURE 1.** Schematic overview of the nomenclature of the major cell types in the CNS. Nomenclature of the glia cell family is sometimes especially confusing. Shadowed boxes are the major cell types in the brain parenchyma (and are the names of the cells that will be referred to in the review). Microglial and endothelial cells are thought to be of mesenchymal origin while the other cell types are of neuroectodermal origin. The choroid plexus may be regarded as a specialized outgrowth of ependymocytes making up the blood-CSF barrier.

and IGFBP-6[131] has also been reported. More specifically, expression of IGFBP-2 simultaneously with IGF-II has been reported in the choroid plexus and meninges[62], and with IGF-I in neuronal structures[57]. A recent study suggests expression of IGFBP-2 in microglial cells and possible involvement in the pathogenesis of multiple sclerosis[191]. Morevoer, IGFBP-2 in particular appears to depress an increase by IGF-I on astrocyte intercellular communication by gap junctions[172], which may have consequences for the repair of lesions in the brain. On the other hand, expression of IGFBP-5 has been shown to be more selectively coexpressed with IGF-I or in the vicinity of IGF-I–producing neurons[53]. IGFBP-6 is synthesized in the choroid plexus and by astroglial cells[95], and recent studies suggest a regulatory role of cell proliferation in the CNS[190]. Although a wide range of actions in both physiology and pathophysiology has been proposed, it is clear that more studies are needed to clarify the exact roles of the different IGFBPs in the CNS (for reviews, see [104,170]), especially in the context of brain injury and repair.

### GH and IGF-I and the Blood Brain Barrier

The transport of GH and IGF-I from the blood to the brain may potentially occur by any of three mechanisms in normal physiology: (1) they may circumvent the blood brain barrier (BBB) as in the circumventricular organs (such as in the median eminence of the hypothalamus); (2) they may actively or passively pass the brain parenchyma capillaries through the BBB (here designated capillary-bed BBB); or (3) they may be filtered through the choroid plexus into the CSF (the so-called blood-CSF barrier). From the CSF, they may either diffuse into the brain parenchyma through the ependymal cells, but they are mostly taken up into the subarachnoid villi and transported away to the blood directly or via the brain lymphatic system. The three mechanisms are sometimes confused with each other, which may explain some of the differences in interpretations of different studies. The transport or leakage from CSF over the ependymal cells both from and into the brain parenchyma intercellular space appears to be a possibility in the case of IGF-I, as exemplified for IGF-I that penetrates up to 1.25 mm into the ventricle walls[215]. Small quantities of brain parenchymal intercellular substances may also be cleared into the blood by the lymphatic system of the brain.

Previously, controversy existed regarding the extent to which GH can cross the actual capillary-bed BBB, but there is now growing evidence of GH uptake from the blood stream into the brain parenchyma through different routes. First, GH-R is present in the choroid plexus and it has been suggested to play a role in the transport of GH across the blood-CSF barrier[37]. Moreover, when GH was administered peripherally to patients with GH deficiency, a tenfold increase in CSF levels of GH was reported[68], which would support passage of GH over the blood-CSF barrier or alternatively another specific transport mechanism for GH from periphery to the CNS. This is in contrast to previous studies using iodinated GH that showed only a limited passage of GH over BBB[6,12], as these studies showed mostly the effect of the capillary-bed BBB. However, a recent study that examined GH absorption via the capillary-bed BBB into both the mouse and rat brain *in vivo* and GH pharmacokinetics of endothelial cell cultures *in vitro* favors the idea of an influx of GH by simple diffusion, despite the absence of a specific transport system[216]. In addition, the BBB (both blood-CSF barrier and capillary-bed BBB) might be compromised in different pathological conditions such as after a hypoxic-ischemic event or even by hypophysectomy itself as previously suggested[124]. Taken together, there is growing support of a passage of GH over the BBB, although the exact mechanisms and extent of passage remains to be further elucidated.

It is now well established that IGF-I reaches both the brain parenchyma and CSF from the peripheral blood stream. From pharmacokinetic studies *in vitro,* it appears that IGF-I uptake, unlike the uptake of GH[216], is mediated by a specific carrier both in the capillary-bed BBB[23] and in the blood-CSF barrier[134,210]. Interestingly, at least in rats, the blood-CSF barrier uptake appears to be saturated at a serum IGF-I of about 150 ng/ml[134], which is lower than the usual range of serum-IGF-I in the normal rat (approximately 1000 ng/ml[122]). Moreover, exercise per se appears to enhance IGF-I transport over the BBB[135], as it appears mostly by the capillary-bed BBB. Also, injuries may enhance IGF-I uptake into the brain parenchyma, possibly by opening of the BBB, as suggested by studies showing that hypoxic-ischemic injury enhances centrally administered $^{3}$H-IGF-I staining in the brain[75].

Thus, although not fully characterized, there appear to be mechanisms for transport of both GH and IGF-I across the BBB.

## EFFECTS OF GH-IGF-I IN THE ADULT BRAIN

### Different Aspects of the Concept of Brain Plasticity

The ability to adapt brain function and cell biology, including structural changes in the brain, is often referred to as the plasticity of the brain. Brain plasticity can be studied using tools to access mRNA, protein, post-translational modifications, cellular and regional expression, protein activity function, or cell physiology (e.g., ions and neurotransmitters, action potentials). In the brain, cellular function can be described in terms of interactions between the major cell types of the brain and the concept of a functional neuron-glia unit, proposed as early as in 1962 by Holger Hydén (Fig. 1)[3]. Cellular morphology may be affected in, for example, dendritic arborization, axon elongation, and astrocyte process extension (Fig. 2). In addition, adult cell genesis (proliferation), including neurogenesis and apoptosis, are processes that may be affected by different stimuli. When these processes are influenced for a longer period of time, macrostructural changes in the brain have been observed in, for example, the posterior hippocampus[138]. Within this concept of brain plasticity, we will try to describe both cellular and functional mechanisms of action for GH and IGF-I in the adult brain.

### Cell Genesis in Relation to Neurogenesis

Adult cell renewal has been established for decades primarily with respect to endothelial cells, microglial cells, and to a certain degree astrocytes (read reactive astrocytosis)[1,2,4], while suggestions of neurogenesis and oligodendrogenesis were ignored until the early 1990s. Particularly important regarding adult neurogenesis were the discoveries of self-renewing cells with multilineage potential in the adult mammalian brain[49,50], the human brain included[98,142]. There are two sites with a high density of proliferating progenitor cells in the adult brain: the subgranular zone (SGZ) of the dentate gyrus of the hippocampal formation[4,78,142], and the subventricular zone (SVZ)[106,109,117]. In the dentate gyrus, the progenitor cells migrate into the granule cell layer where they differentiate into granule cell neurons. In the SVZ, the progenitors migrate through the rostral migratory stream to the olfactory bulb where they differentiate into interneurons, but also into oligodendrocytes and astrocytes[214]. However, cellular proliferation also occurs at a much lower frequency in brain regions outside of the two brain sites described. The status of adult cellular proliferation in, for example, the cerebral cortex has not been completely elucidated. A resident population of dividing cells in the adult cerebral cortex has been suggested[120], but there may also be an ongoing migration of newborn cells coming from the SVZ[114]. Altogether, there seems to be two major regions with ongoing adult cell proliferation in the brain (including neurogenesis), but there is also evidence of a more-dispersed cell proliferation (normally without neurogenesis) at a much lower rate outside of these two major sites.

### GH and IGF-I and Cell Genesis

#### *Trophic Effects in the Growing Brain*

In 1968, a study indicated that GH had trophic effects in the CNS. The study reported that treatment with GH increased the thickness of diencephalic structures (e.g., the anterior and posterior commisure) in the growing postnatal brain[5]. Later, studies of Little mice (deficient in serum GH and IGF-I due to a mutation in the GHRH-receptor) showed that these substances are important for cell proliferation and for oligodendrocyte parameters in the developing brain[28]. Similarly, it has been shown that treatment with GH antiserum causes a decrease in proliferation and myelination in growing rats[8]. Conversely, GH overexpression increases brain weights and the astrocyte intermediate filament glial fibrillary protein (GFAP)[69]. There are data in the developing animal that indicate that GH affects both neuron and astrocyte proliferation. The suppressor of cytokine signaling-2 (SOCS-2) is held to be an inhibitor of GH signaling; therefore SOCS-2–disrupted mice are hyper-responsive to GH[200]. In these mice, grown to a young adult age, there were fewer neurons and more astrocytes in several layers of the cerebral cortex and in the striatum, although the hypothalamus was unaffected[200]. Conversely, the neuron-to-glia ratio increased in GHR -/- mice[200]. This was also shown in embryonic progenitor cultures taken from the SVZ, where GH decreased neuronal differentiation[169]. In addition, dendritic branching decreased in pyramidal cells and neuronal somas were also decreased in size[200]. Also, it seems that GH overexpression increases GFAP content in the brain[69], indicating an effect on astrocytes. Surprisingly,

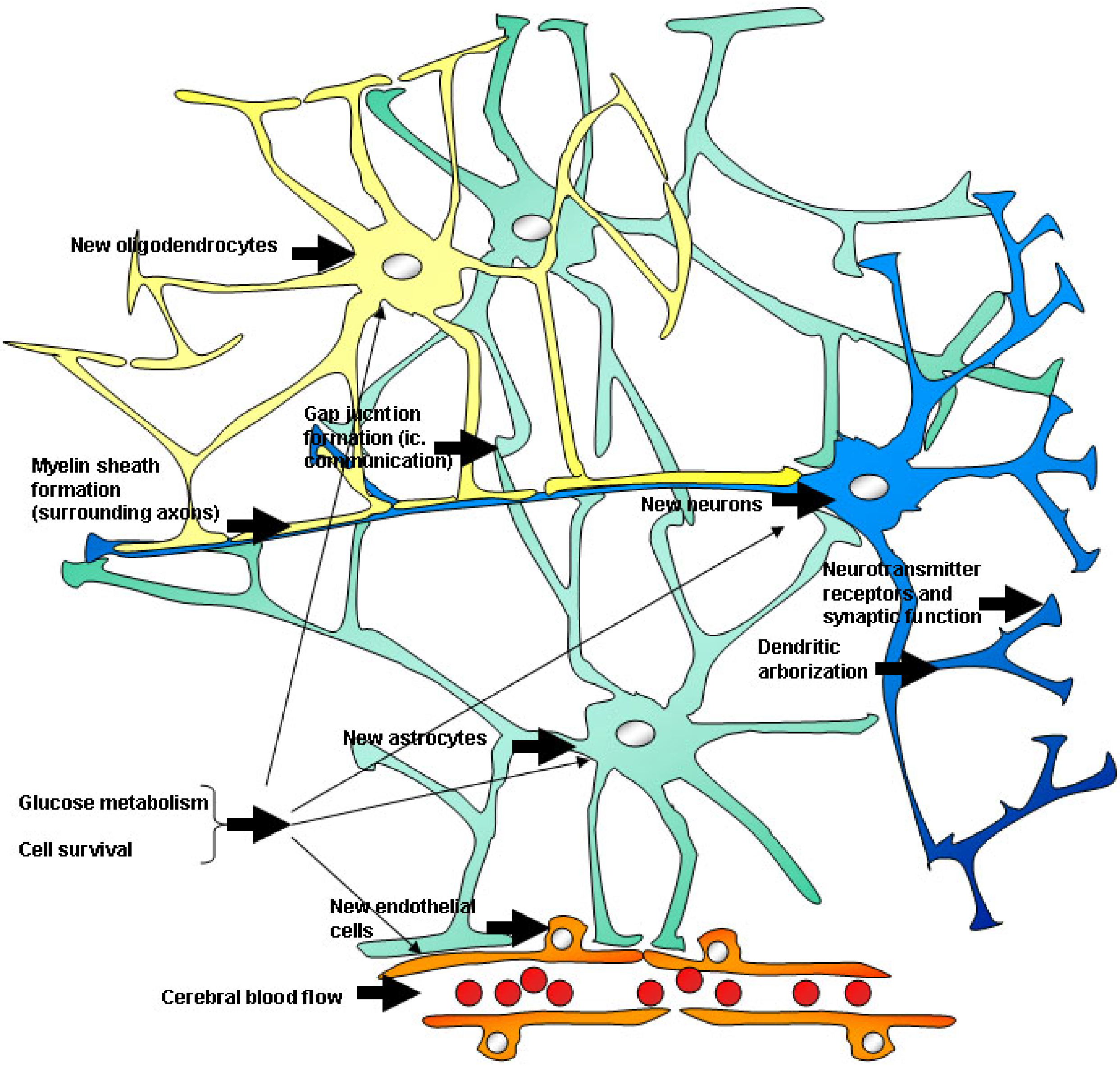

**FIGURE 2.** Schematic overview of the cellular interactions through which GH and IGF-I act. Yellow = oligodendrocyte; blue = neuron; green = astrocyte; red = blood vessel and erythrocytes. Arrows indicate the cellular targets that are affected by GH and IGF-I. Details are comprehensively described in the main text.

GH increases both neuronal and glial relative cell numbers in embryonic cultures from cerebral cortex[173], which may indicate a partly different effect of GH on the embryonic brain compared to what happens later during early postnatal development.

IGF-I overexpression induces even more pronounced effects. For example, it has been found that brain size *in vivo* increases by 55% and myelin content increases by 130% at 55 days of age[55]. Surprisingly, oligodendrocyte cell density was unchanged. Furthermore, transgenic IGF-I overexpression has been shown to promote neurogenesis and synaptogenesis in the hippocampal dentate gyrus during postnatal development[141]. Moreover, IGF-I overexpression results in increased neuron number and growth in the medullary nuclei of the mouse[108] and promotion of neurogenesis and synaptogenesis in the hippocampal dentate gyrus during postnatal development[141]. Conversely, in IGF-I knockout mice, hypomyelination, reduction of granule cells in the hippocampus, reduced neuron and oligodendrocyte numbers within the olfactory bulb, and reduction of brain total size are found[65,96]. These findings have parallels in different *in vitro* situations. For example, IGF-I is a mitogen for embryonic progenitors[22,82] and is needed for the propagation of fetally derived neurospheres[150] and neuroblast cultures[54]. To summarize, the above *in vivo* studies have focused on the consequences of GH/IGF-I deficiency (both local and systemic deficiency) on cell genesis during development and young ages, whereas the *in vitro* studies may largely be regarded as investigations of embryonic local (paracrine/autocrine) effects of GH/IGF-I. However, IGF-I is also known to affect cell genesis in the *adult* brain, as will be discussed in the following sections.

### *Serum-Derived GH and IGF-I and Adult Cell Genesis*

The extracerebral and peripheral increases of GH and IGF-I in adult animals are part of a physiological response to exercise and, thus, are of interest as GH or IGF-I may be used as exogenous pharmacological agents for enhancing cell genesis in the CNS.

The investigation of proliferation and differentiation of progenitor cells in the adult brain has been performed in hypophysectomized rats[132], liver-specific IGF-I knockout mice treated with *peripherally* administered IGF-I[196], or in normal rats with an antibody blockade of peripheral IGF-I with antibodies[159]. In these paradigms, bromodeoxy uridine (BrdU) has been used to monitor proliferation in combination with double/triple immunofluorescence for cell-specific markers in the evaluation of cell fate differentiation.

#### The Two Proliferative Zones in the Adult Brain

##### *The Hippocampus — New Neurons, Oligodendrocytes, and Endothelial Cells*

Peripheral IGF-I in young adult rats, either hypophysectomized or intact, has been shown to increase both cellular proliferation in the dentate SGZ and the subsequent migration and differentiation of progenitor cells within the granule cell layer (GCL) of the dentate gyrus in the hippocampus[132,196]. IGF-I increased BrdU-positive cells by about 80% in the GCL after 6 days of administration and after 20 days of IGF-I treatment, approximately 50% of the increase of BrdU-positive cells remained[132]. This likely reflects both apoptosis and cellular migration away from the site of genesis (for more details on this topic, see [205]).

Peripheral rhIGF-I treatment for 20 days in hypophysectomized rats has been shown to increase both BrdU-positive cells and the fraction of newly generated neurons in the GCL as evaluated by the neuronal markers Calbindin $D_{28K}$, microtubule-associated protein-2 (MAP2), and NeuN[132]. However, IGF-I did not affect the fraction of newly generated astrocytes[132]. The fact that there was a positive correlation between body weight gain (by IGF-I treatment) and the number of BrdU-positive cells in the GCL further underlines that peripheral IGF-I affects the degree of hippocampal proliferation. Overall, the number of new neurons increased 78 ± 17% more after 20 days in IGF-I–treated animals[132]. This is a relatively larger increase than the number of surviving cells per se. Increased circulating IGF-I induced by exercise elicits similar effects on hippocampal neurogenesis[159]. It appears that circulating IGF-I also directly affects angiogenesis in the hippocampus, as evidenced by increased numbers of BrdU-positive cells, which are also positive for lectin (an endothelial marker)[196]. Furthermore, it appears that local IGF-I also has relevance for hippocampal neurogenesis because in GH-deficient long-lived Ames dwarf mice a combination of increased local IGF-I and increased neurogenesis was observed, despite low levels of circulating IGF-I[219]. Finally, it was recently shown that IGF-I has an instructive role inducing oligodendrocyte cell fate from adult hippocampal progenitor cells *in vitro* and increasing oligodendrocyte markers in the hilus region of hippocampus *in vivo*[194]. It appears that IGF-I preserves its capacity to induce adult hippocampal proliferation of cells after ischemic injury[178]. The functional benifit of this remains to be established.

The action of IGF-I on hippocampal neurogenesis may require permissive conditions provided by other agents such as the estrogen receptor system. This is suggested by findings that the estrogen receptor antagonist ICI 182780 blocks the intracerebroventricularly IGF-I–induced neurogenesis in the dentate gyrus, whereas coadministration of subcutaneous estradiol and intracerebroventricular IGF-I enhances neurogenesis[185]. Estrogen receptors both of $\alpha$ and $\beta$ types are widely expressed in the hippocampus and elsewhere in the brain of animals of both sexes (for review, see [144]). IGF-I and estradiol also have permissive and additive effects for other processes in the brain, e.g., synpatogenesis and neuroprotection (for review, see [144]). Interestingly, it appears that estradiol may have similar neuroprotective effects in both female and male rats[220].

In summary, there is evidence that IGF-I is one of the major peripheral stimuli for hippocampal cell genesis and neurogenesis. There is also support, however, for other peripheral factors (e.g., the estrogen system) that may interact with the effects of IGF-I.

#### *In Vitro* — Cells Derived from the Adult Hippocampus

Adult hippocampal progenitor cells (AHPs) derived from the adult rat hippocampus are thought to correspond to progenitor cells in the adult SGZ. The AHP cells express IGF-I-R, IGFBP-2, and IGFBP-4[172], which is probably similar to conditions *in vivo*, as IGF-I-R appear to be highly expressed in the SGZ of the hippocampus[51,162]. IGF-I treatment increases the amount of IGFBPs, while FGF-2 pretreatment increases the amount of IGF-I-R protein, which may explain the positive additive effect of FGF-2 and IGF-I on AHP cell proliferation[172]. Using inhibitors and dominant negative constructs, it appears that the MAPK (mitogen-activated protein kinase) signaling pathway is required for IGF-I–stimulated proliferation in adult progenitor cells[172]. The proliferative effect is separated from the effects mediated by insulin, as shown in dose-response experiments with high or low concentrations of insulin. The phenotypic distribution after 10 days of IGF-I treatment *in vitro* shows that the fraction of newly generated neurons increases significantly. These results suggest that the proliferative effects of IGF-I on AHPs found *in vivo* are also present *in vitro* and that they are mediated by the MAPK signaling pathway.

#### Potential Effect in the SVZ and the Olfactory Bulb

The SVZ is the other main site of cell division that persists throughout adulthood. Migration of cells occurs mainly through the rostral migratory stream (RMS) to the olfactory bulb. In the olfactory bulb, the cells mature into interneurons, but also into astrocytes and oligodendrocytes. Although not well understood, there are data supporting an additional migration of cells from the SVZ into the cerebral cortex[114,120,161]. As yet, no study has examined the effects of IGF-I on adult proliferation in the SVZ. However, during the development of the olfactory bulb, IGF-I knockout animals show an abnormal formation of the olfactory bulb mitral cell layer and they also have altered radial glia morphology[187]. As SVZ is the source of olfactory neurons, also during development, it appears that IGF-I has the potential to affect SVZ proliferation. This is supported by the fact that IGF-I is highly expressed[41] and the IGF-I-R is moderately expressed[51] in these cell layers.

In the olfactory epithelium (part of the peripheral nervous system) and within the olfactory bulb (part of the CNS), neurogenesis persists into adulthood[67]. The adult olfactory bulb is the target for most cells migrating from the SVZ. It appears that type A cells within the SVZ are the source of migrating neuroblasts[179], which arrive to the olfactory bulb for maturing locally[176]. Although their origin is from the SVZ, it also seems that some precursor cells continue to divide *in situ* within the olfactory bulb[164]. Furthermore, IGF-I stimulates proliferation in adult glial olfactory cultures[160] and certainly appears to affect neuronal axonal extensions and glial morphology in organotypic cultures[63]. It thus seems plausible that peripheral IGF-I affects adult cell genesis in the adult olfactory system.

Altogether, available data support the idea that IGF-I increases adult cell genesis in the SVZ, as well as migration into the olfactory bulb. However, the nature and functional significance of these events remains to be investigated.

### Neuroprotective Actions of the Somatotrophic Axis

#### *Neuroprotection and Neuroregeneration*

The research on acute treatment of stroke has largely focused on the effects of various neuroprotective agents in sparing the neurons in the penumbra zone of an ischemic lesion. Several such compounds, including IGF-I and GH, have been successful in reducing the damage after ischemic lesions in experimental animal models. These substances may potentially also have protective effects in humans. However, clinical studies are needed to confirm this issue.

After the acute phase in an ischemic lesion, which may last for days in humans, there is a recovery phase where patients usually regain some lost abilities and skills. As long as the ischemic pathology proceeds, one can administer a compound to act in a manner of "neuroprotection", i.e., to save tissue and cells from dying. This is largely a different process than the much longer regenerative plasticity that takes place during recovery after an ischemic injury. A compound can thus be stated to act in a manner of neuroprotection or neuroregeneration (for review, see [205]). Of course, the two processes may be affected simultaneously by a given substance, as is probably the case for GH and IGF-I. Recovery is also often enhanced by active rehabilitation programs. Interestingly, this may have a parallel in the situation where several plasticity mechanisms including neurogenesis are observed in rats and mice living in enriched environments[100,121], learning tasks[113], and physical activities[129].

#### *Neuroprotective Effects of IGF-I*

IGF-I, IGFBP-2, and IGFBP-3 mRNA expression have all been shown to increase after hypoxic-ischemic injury (HI)[45,52,115] and it has been speculated that the increased IGF-I expression may be an endogenous mechanism to reduce the injury after an insult[79,95]. Moreover, since the early 1990s, intervention studies have shown neuroprotective effects of IGF-I in different models of injury and insults to the brain. IGF-I has been administered through different routes, for example, directly through intracerebroventricular administration[56,209]. However, similar effects have also been found after peripheral administration of IGF-I[93,110]. Interestingly, it has also been shown that elevated IGF-I after exercise may mediate neuroprotective effects against several injuries in the adult rat brain[151].

Initial studies were focused mainly on the tissue-protective aspects of IGF-I administration. These effects can largely be linked to antiapoptotic mechanisms[181,209]. IGF-I has been shown to promote cell survival and inhibit apoptosis *in vitro* in a number of cell types including neuronal cells[102,107, 127,148]. Studies performed *in vivo* have demonstrated neuroprotective effects after HI insult in adult rats and fetal lambs, reducing both the loss of neurons and demyelination[56,77,153]. The signaling of the survival-promoting effects of IGF-I have been studied mostly *in vitro* and are considered to mainly occur through the PI3K (phosphoinositide-3 kinase)/Akt (a kinase and a transcription factor also known as protein kinase B, PKB) pathway or through activation of the MAPK/Erk (extracellular signal-regulated kinase) pathway[84,90,102,123,149](Fig. 3). A recent *in vivo* study showed that intracerebroventricular administration of IGF-I for 3 days after the insult reduced HI brain injury by 40% compared to controls and that the neuroprotective effect was accompanied by increased levels of phosphorylated Akt and Erk, inactivation of the proapoptotic glycogen synthase kinase kinase 3 β (GSK3β), and with reduced caspase-3– and caspase-9–like activity[209]. Also, the neuroprotective effect of IGF-I has been shown to synergize with erythropoietin in a model of N-methyl D-aspartate (NMDA)–induced insult[192]. However, it is likely that IGF-I simultaneously acts at several levels of cell turnover including both

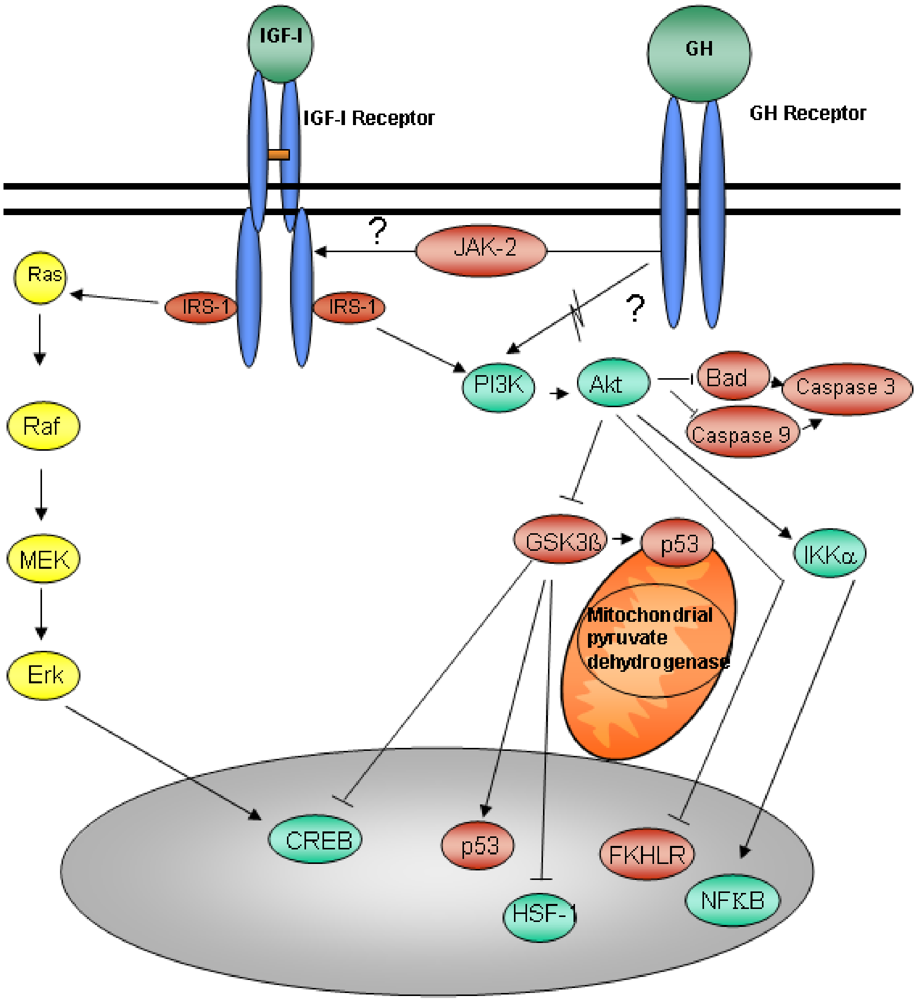

**FIGURE 3.** Proposed signaling pathways for IGF-I and GH with regard to cell survival. IGF-I binding to its receptor leads to activation of the PI3K/Akt or MAPK (mitogen-activated protein kinase = extracellular signal-regulated kinases, ERK) pathways. Both pathways can stimulate cell survival through activation of antiapoptotic substrates (green) such as CREB, HSF-1, IKKα, and NFKB transcription factors involved in cell survival. PI3K activation of Akt can also lead to inhibition of proapoptotic substrates (red) as GSK3β, Bad, FKHLR, p53, caspase-3, and caspase-9. The proapoptotic agent GSK3β acts by promoting actions of both nuclear and mitochondrial p53, which lead to release of cytochrome C and caspase-3 activation. Although GH-R–mediated cell survival pathways remain more uncertain (as indicated by question marks), we have included potential pathways as recent data indicate that GH may activate at least the PI3K/Akt pathway at the skeletal muscle (for review, see [211]). Another recently shown possibility in a preadipocyte cell line is that GH-R cross-activates the IGF-I-R directly via JAK-2[195]. These possibilities remain unproven for the CNS, but likely some of these mechanisms may be active and explain the manifold interactions in the effects of GH and IGF-I in the brain. Abbreviations: PI3K (phosphoinositide-3 kinase), MAPK (mitogen-activated protein kinase), Erk (extracellular signal-related kinase), CREB (cAMP-response element binding protein), HSF-1 (heat shock factor 1), IKKα (I-kappa-B kinase-alpha), JAK-2 (Janus kinase 2), NFKB (nuclear factor kappa B), GSK3β (glycogen synthase kinase kinase 3 beta), FKHLR (forkhead transcription factor). MEK is an MAP or ERK kinase (also known as Mitogen-activated protein kinase kinase, MAPKK). Ras, a GTB-binding protein, the gene origainlly named after rat sarcoma. Raf=Ras-activating factor.

protective (cell survival) and regenerative (cell genesis). Both processes could then improve the final functional outcome after an injury.

#### *Growth Hormone*

Only a limited number of studies have addressed possible neuroprotective effects after administration of GH. When administered subcutaneously to neonatal and juvenile rats in high doses, a neuroprotective effect was observed[115,158] accompanied by antiapoptotic actions[202]. However, when GH was given peripherally, in lower and more physiological doses, no statistically significant neuroprotective effects were achieved in rats[124]. It appears that GH treatment spares neuronal loss after HI injury primarily in the frontoparietal cortex, hippocampus, and dorsolateral thalamus, but not in the striatum. The spatial distribution of the neuroprotection by GH correlates with GH-R distribution, but not with the IGF-I-R distribution[158]. This indirectly indicates that the neuroprotective effects of GH may be mediated through activation of the GH-R without involving the IGF-I-R, thereby implying that GH has neuroprotective effects per se. It thus appears that GH has some neuroprotective effects on its own, independent from IGF-I.

#### *GH vs. IGF-I Effects*

IGF-I has been studied more extensively than GH in terms of adult brain cell proliferation and cellular differentiation. The most important reason for this is that whenever GH is given systemically, IGF-I will also be released into the circulation from the liver[19] and be produced locally in the brain[19,80,163].

Therefore, different approaches are needed to study different aspects of the effects of GH and IGF-I on the brain. First, studies of short-term effects of GH may often be specific for GH because IGF-I would not have had time to be induced. However, that reduces the time window for studying acute effects of GH (less than hours). A second alternative is to study GH (also inducing liver IGF-I systemically) and IGF-I (slightly decreasing systemic GH due to feedback inhibition) treatments and compare their relative effects[83]. If this is done in hypophysectomized rats, IGF-I treatment will not cause a feedback inhibition of systemic GH as it is already depleted[133]. In this way, a more distinct IGF-I response can be compared to the combined effect of IGF-I and GH, which is the effect of GH treatment. The third way to study the effects of GH would be to use antibodies or other IGF-I blockers. A fourth approach would be to use transgenic constructs, either disrupting liver IGF-I or brain IGF-I functions or both. Liver IGF-I knockout mice are available, but to our knowledge they have not been used for assessing GH effects. A fifth approach is to use transgenic constructs with disrupted GH signaling, as for example GH-R disruption[200] or SOCS-2 disruption[169,200], preferably in adult conditional transgenic animals. However, at present, there is only one liver-IGF-I conditional adult or young adult knockout animal model[125,196].

In summary, distinguishing the effects of GH and IGF-I on the brain is an important but difficult task, especially in the light of potential cross-activation of the receptors (as suggested in Fig. 3). Different conditional transgenic constructs may potentially aid these investigations.

### Miscellaneous Effects in the Adult Brain

#### *Neurotransmitters*

As GH is reportedly known to exert effects on the brain, one line of research has sought to evaluate how neurotransmitters and neurotransmitter receptors are affected. Altogether, it appears that GH, and to some extent IGF-I, affects most of the major neurotransmitter differently in several brain regions, including the monoamine (serotonin, noradrenaline = norepinephrine) system, the dopaminergic system, the glutamatergic system (at least at the receptor level), the opioid system, and the cholinergic system.

Interestingly, it has been shown that GH affects the monoamine levels within 15 min after intraperitoneal administration in rats[7] and its effects are different in normal and hypophysectomized rats. In normal rats, GH decreases the levels of both 5-hydroxyindoleacetic acid (5-HIAA) and norepinephrine in the diencephalon and brainstem, without affecting telencephalic concentrations. In hypophysectomized rats, however, GH produces significant elevations of norepinephrine and 5-HIAA levels in all brain regions[7].

Similarly, bGH transgenic mice evidence increased tissue levels of serotonin and 5-HIAA acid in several brain regions[126]. Although the two experimental paradigms, using replacement treatment or chronic overexpression of GH, are difficult to compare directly, they nevertheless show similarities with respect to the serotonin system. However, these results do not extend to analysis of human CSF as the norepinephrine metabolite 3-methoxy-4-hydroxyphenyl-ethylene-glycol and the serotonin metabolite 5-HIAA are not affected after GH treatment[68]. The different results of the monoamine metabolites may be due to the only partial communication existing between the CSF and the brain parenchymal extracellular fluid since the CSF is usually reabsorbed via the subarachnoid villi or to a minor degree drained via the brain lymphatic system (see section on "GH and IGF-I and the Blood Brain Barrier").

GH has been shown to affect the dopamine system in several ways. First, it reduces dopamine (DA) release in the rat subependymal layer and median eminence after intravenous administration[14]. This was interpreted as underlying the feedback loop of GH on its own release by reducing DA synthesis and release in the median eminence leading to increased somatostatin release[14]. Second, bGH transgenic mice display decreased DA levels in the brain stem and decreased levels of the DA metabolite 3,4-dihydroxyphenylacetic acid in the mesencephalon and diencephalon. The altered DA levels may be a neurochemical correlate of the altered spontaneous locomotor activity. The prolonged increased spontaneous locomotor activity may, in turn, reflect a disturbed habituation process, as that locomotor activity normally should decrease after some time[126]. Similarly, dopamine metabolite homovanillic acid decreases in the CSF on GH treatment in GH-deficient humans[68].

The glutamatergic receptor system is present throughout the brain in both neurons and astrocytes. Three major types of receptors are known, one of these being the NMDA receptor. The NMDA receptor subunits NR1 and NR2B are thought to be involved in memory formation in the hippocampus. Therefore, it is interesting that GH appears to affect these transcripts. GH treatment in young adult hypophysectomized rats decreases the hippocampal mRNA expression of NR1, but increases the NR2B subunit[166], whereas in elderly rats, GH increases both the NR1 and NR2A transcripts. Similar results have been obtained with IGF-I therapy[213]. The results are particularly interesting as it appears that the ratio of NR2B to NR2A mirrors the potential for synaptic plasticity. However, there remains much to be investigated due to complexity of the glutamate receptor system, involving several additional subtypes of receptors, different expression in both cell type–specific and layer-specific fashion, and brain region heterogeneity.

GH treatment of GH-deficient humans slightly decreases the CSF vasoactive intestinal peptide (VIP) and increases CSF immunoreactive beta-endorphin[68]. Presumably, this is one possible correlate of the state of well being associated with GH treatment in these patients[68]. Often, an increase in a given agonist is reflected in an inverse decrease in the corresponding receptor density. This is likely the case with beta-endorphins and one of their receptors the delta-opioid receptor. On GH treatment in hypophysectomized rats (known to increase beta-endorphins), a two- to threefold down-regulation of delta-opioid receptor densities in the cerebral and cerebellar cortex is observed[186]. Somewhat surprisingly in the Johansson study, there were no significant changes in the CSF concentrations of other parts of the opioid system, such as in the enkephalins and dynorphin A[68]. Neither were gamma-aminobutyric acid, somatostatin, or corticotropin-releasing factor affected[68]. However, as mentioned, it is difficult to compare different methods and experimental paradigms (see also section on "GH and IGF-I and the Blood Brain Barrier"). Furthermore, the results may also be influenced by the fact that some of the neuropeptides are thought to be sensitive to correct handling and analysis (personal communication by Prof. Fred Nyberg, Uppsala University and Dr. Jan-Ove Johansson, Göteborg University).

Finally, it appears that the cholinergic system has functional links with GH and IGF-I. First, it seems that functional stimulation of muscarinic transmission by the cholinesterase inhibitor pyridostigmine may enhance GH release from the pituitary under certain conditions[33,44]. Second, IGF-I affects potassium-evoked acetylcholine (ACh) release in tissue slices of the adult hippocampus and cerebral cortex. In slices from the adult hippocampus, IGF-I decreases the release of ACh[25]. In slices of the adult cerebral cortex (*parietal*), however, IGF-I appears to increase the release of ACh at a defined concentration window[59]. Likely, much of the effect in the slices from the cerebral cortex was mediated by the N-terminal tripeptide of IGF-I: glycine-proline-glutamate (GPE), because this substance enhanced ACh release to a greater degree and truncated IGF-I (i.e., IGF-I without the N terminal tripeptide GPE = des-IGF-I) had no effect on ACh release[59].

In summary, there is plenty of evidence that GH and IGF-I affect several of the major neurotransmitter systems. Many of these effects act directly via neurons and can be observed after only a short exposure. However, some of these effects also interact with the other main cell types of the brain, the glial cells, as will be touched on below.

### Intercellular Communication in Astrocytes

In recent years, astrocytes (see Fig. 1) have gradually attracted more interest as being active participants in brain energy metabolism and in regulating the synaptic efficacy by, for example, modulating glutamate neurotransmission at the synapse (for review, see [174]). One of the specialized functions of astrocytes that enables the cells to perform these processes is the functional syncytium that couples astrocytes to each other. These are made up of intercellular pores between the astrocyte cell membranes that allow the cells to transmit low molecular (<1000 Da) compounds from cell to cell for quite some distances. Sometimes this ability of communication is referred to as an "astrocyte syncytium"[15,30]. The intercellular pores are structurally organized as gap junctions made up of two sets of hexameric connexin proteins. In the adult brain, astrocytes almost exclusively express the subtypes connexin30 and connexin43, while some neurons express lower amounts of connexins (for review, see [197]). It appears that GH and IGF-I affect the abundance of connexin43. *In vivo*, systemic GH increases connexin43 in hypophysectomized rats, while systemic IGF-I does not[133]. *In vitro*, IGF-I increases both connexin43 protein and intercellular communication of astrocyte cultures, while GH does not[172]. The results have significance from two perspectives (for review, see [204]). First, the GH and IGF-I system appears to be involved in the astrocyte communication system, which probably reflects parts of the neurocognitive effects of GH and IGF-I (especially considering the functional neuron-glia synaptic interplay). As several of the neurotransmitters (e.g., glutamate, aspartate[16]) and glucose[40] may be taken up by astrocytes and can pass from astrocyte to astrocyte via gap junctions[11], there is here a potential of a further level of complexity in transmitting signals in the CNS. Second, the results indicate that local brain IGF-I may mediate effects of systemic GH in terms of astrocyte expression of connexin43. However, further studies are needed to elucidate the exact roles of local and systemic GH/IGF-I with regard to gap junctions in the brain.

### Energy Metabolism in the Brain

In the brain, both astrocytes and neurons take up glucose from the bloodstream via endothelial cells at the BBB. From there, glucose is transported either by diffusion or active transport into the brain parenchyma. However, only astrocytes have the ability to both store and break down glycogen and form lactate[94] and transport either glucose[81] or lactate[86] intercellularly by gap junctions, and thus direct the supply of energy to neurons. One estimation of brain energy expenditure in gray matter states that about 85% of energy is used by neurons[199]. However, in situations with high activity, neurons preferentially feed on lactate, which is produced by astrocytes from glucose and glycogen in response to neuronal glutamate[199]. Interestingly, the IGF-I system appears to affect glucose metabolism in brain cells by several mechanisms.

First, under different conditions, IGF-I appears to enhance glucose utilization in both ependymocytes (see Table 1 and Fig. 1) and neurons. IGF-I appears to stimulate ependymocyte glucose uptake ten times more potently than insulin, likely via the glucose transporter GLUT1[203]. Also, neurons may take up glucose in response to IGF-I, as 2-Deoxy-d-[1-(14)C]glucose is significantly reduced in synaptosomes prepared from IGF-I -/- brains, and the deficit is overcome by IGF-I[136]. Moreover, astrocytes appear to be affected by IGF-I with respect to glucose uptake, at least transiently under development[40].

Second, one of the mechanisms for the neuroprotective and antiapoptotic effects of IGF-I may be that of enhancing glucose uptake in neurons exposed to glucose deprivation[201]. Also, the enhanced glucose uptake ability into brain cells, in addition to stimulating the IGF-I system, may be one of the mechanisms by which estradiol acts in a neuroprotective manner[152]. For example, it was shown that estradiol treatment induces a two- to fourfold increase in GLUT3 and GLUT4 mRNA (mainly expressed in neurons) levels and lesser, but significant, increases in GLUT3 and 4 protein levels. Estradiol treatment also induces an approximately 70% increase in parenchymal GLUT1 mRNA levels (capillaries and glial cells), but does not appreciably affect vascular GLUT1 gene expression. Furthermore, IGF-I mRNA levels have been found to significantly increase in estradiol-treated animals, but IGF-I receptor mRNA levels were not altered by hormone treatment[152]. Interestingly, it appears that IGF-I may restore the loss of glucose utilization that occurs in the aging brain. Administration of IGF-1 to aged animals has been found to increase rates of glucose utilization by 11–14% in the anterior cingulate of the cortex, CA1 region of the hippocampus, and the arcuate nucleus of the hypothalamus[156].

Altogether, it appears that IGF-I affects glucose metabolism in both astrocytes and neurons in the adult brain. However, more data are needed to clarify the exact mechanisms with regard to functional parameters such as learning. On these lines, it will be interesting to await data as to whether GH has any direct effects. To our knowledge, no study has addressed this issue to date.

### Neuronal Dendritic Arborization — Electrophysiology

Both episodic (≈ declarative including verbal, conscious) and nonepisodic (≈ nondeclarative including nonverbal, habitual, and usually nonconscious) memory imprinting is largely attributed to neuronal synapse remodulation described both by electrophysiological events and by structural plasticity of axonal-dendritic arboreal trees. In terms of electrophysiology, especially long-term potentiation (LTP) of synaptic strength is thought to mirror memory imprinting, e.g., both in the hippocampus and in the cerebellum.

GH and IGF-I appear to affect neuronal dendritic tree complexity as well as electrophysiological aspects of neuronal function. IGF-I affects dendritic arborization during the development of cerebellum[89,182] and in the developing adult cerebral cortex studied by *in situ* slice preparations[140] or by different models of IGF-I deficiency[17,177]. In mice with IGF-I overexpression, synaptic densities per neuron increase continually with postnatal age in the hippocampus[141]. GH also appears to affect neuronal dendritic branching in the cerebral cortex. Transgenic mice that are either GHR -/- (*hypo*-responsive to GH) or SOCS2 -/- (*hyper*-responsive to GH) both show sparser dendritic branching of pyramidal neurons in the cortex compared to wild type mice[200]. This would somewhat surprisingly indicate a similar effect on dendritic branching in a situation with *either* excess or lack of GH signaling. However, the phenomenon with a similar effect of GHR -/- and SOCS-2 -/- did not extend to other parameters in the same study (e.g., astrocyte and neuron numbers[200]), and the effect may have been secondary to other factors (e.g., not correcting for more neurons in the GHR -/- mice). In addition, as in many other studies, the effect may have been due to IGF-I alterations. The effect of the GH-IGF-I system on dendritic arborization may also have links to the environment as relatively similar and corresponding findings are described after living either in a deprived environment[26] or in an enriched environment[18].

IGF-I has also been administered in a few studies evaluating different electrophysiological responses. In the hippocampus, des-IGF-I has been shown to increase the field excitatory postsynaptic potential (fEPSP) slope, appearing to be mediated through a postsynaptic mechanism involving alpha-amino-3-hydroxyl-5-methyl-4-isoxazolepropionate (AMPA), but not NMDA receptors[218]. Similar effects of IGF-I application have been shown on brain-stem neurons[184]. In the cultures of the developing cerebellum, however, the electrophysiological response by IGF-I on Purkinje cells was scarce, although dendritic arborization outgrowth was clearly stimulated by IGF-I application[180].

In summary, it appears that both GH and IGF-I affect synaptic function either ultrastructurally or by electrophysiological means. However, in this regard, IGF-I has been studied more and it mostly remains to be investigated whether GH affects ultrastructural and functional synaptic formation independently from IGF-I.

#### Cerebral Blood Flow

Neuronal cerebral function is dependent on glucose and on instant continuous oxygenation, both supplied by an uninterrupted blood flow. Cerebral blood flow is relatively easy to study both as a functional parameter, but also in terms of ultrastructural angiogenesis.

It appears that both GH and IGF-I are associated with angiogenesis/blood flow in various areas of the brain. GH treatment increases cerebrocortical arteriolar density in aged rats and, interestingly, the density was correlated to serum-IGF-I levels[92]. In addition, IGF-I increases BrdU-lectin brain vessel density in the cerebellum and hippocampus[196]. There are also some findings that indirectly suggest that IGF-I actually increases cerebral blood flow. First, it has been shown that cerebral arteriolar resistance is decreased by IGF-I in short-term treatment[87]. Second, the same study reported a nonsignificant trend towards an increase in cerebral blood flow[87]. Furthermore, in a study of an elderly human cohort with a high serum-IGF-I, there was an association with an increase in cerebral blood flow in the left premotor cortex (for recalling easy items) and left dorsolateral prefrontal cortex (recalling difficult items) compared to a low serum IGF-I cohort[207]. Collectively, studies on animals and humans indicate that GH and IGF-I have effects on both angiogenesis and cerebral blood flow.

### FUNCTIONAL ASPECTS OF GH-IGF-I IMPACT ON THE BRAIN

#### Aging

The GH-IGF-I system clearly appears to have significance in the aging brain. The increase of IGF-I on cell genesis, including neurogenesis, continues well into middle age[141] and senescence[155]. The serum IGF-I also appears to have a correlation to cognitive memory capabilities in elderly people[87] and in aged animals[101]. Furthermore, GH-IGF-I increases glutamate receptor densities in aged rats[145,166] and there is evidence that GH-IGF-I may ameliorate age deficits in glucose utilization[156] and cerebral blood flow[92,207]. Altogether, GH-IGF-I probably continues to affect the aging brain positively. It will be exciting to await forthcoming studies showing when and where there is a benefit to treat elderly patients. From this perspective, it has been argued that the age-related decrease in GH release (=somatopause) per se would not be an indication for GH treatment[130]. Also, the recent reports that GH is illegally used as an antiaging agent[217] have weak scientific support. Instead, extended use of GH likely shortens life-span as indicated by experiments with transgenic mice[175,208]. The discussion of the pros and cons of GH treatment for use in elderly patients must instead take into consideration that there may be subgroups of patients that would benefit from GH administration for defined periods of time.

#### Stress, Depression, Sleep

Indirect evidence supports the notion that stress and depression inhibit neurogenesis in the adult hippocampus. There seems to be a complex relationship during depression, with increased glucocorticoids, decreased serotonin, and subsequent decreased hippocampal neurogenesis[165]. The inhibitory effects of depression on granule cell production may be prevented by serotonin 1A receptor agonists[112,139]. Depression also decreases the normal GH secretion peak at night[103] and a serotonin antagonist could mimic the effects, causing a significant decline in serum GH concentration[9]. Moreover, serotonin has been shown to stimulate IGF-I release in different non-neural cell systems[91,118]. Hippocampal *atrophy* is found in humans with (1) Cushing syndrome, which is characterized by a pathological hypersecretion of glucocorticoids; (2) episodes of repeated and severe major depression, which are often associated with increased levels of glucocorticoids; and (3) post-traumatic stress disorder (for reviews, see [143,157]). Thus, it may be that, as in rodents, prolonged stress or prolonged exposure to glucocorticoids has adverse effects on the human hippocampus and probably depresses memory capacity[193].

In depression, both the decreased peak serum level of GH (after sleep onset[21,103]) and the frequently increased serum glucocorticoid levels[143] may likely result in decreased IGF-I levels. Reduced IGF-I may be part of the biochemical cascades occurring in association with depression. This is supported by a study of intracerebroventricular administration of IGF-I that mimics the behavioral response of serotonin reuptake inhibitors agonists[212]. These effects may be due to the shared ability of either IGF-I[132,159] or serotonin[105,183,189] to increase hippocampal neurogenesis. Furthermore, *in vitro*, serotonin has been reported to activate IGF-I synthesis[154] as well as brain-derived neurotrophic factor (BDNF)[167]. Although the definite causality of depression and hippocampal plasticity and neurogenesis is unclear, present data suggest there is a coupling that perhaps is also mirrored in the burnout syndrome[193].

Another area in which GH secretion has been investigated is sleeping patterns. It appears that in men suffering from major depressive illness, GH is hypersecreted during wakefulness and the major pulse occurs before sleep onset[21] and with considerabe reduction of peak serum concentration[103]. It is not known whether the altered GH response per se exacerbates or is only a cause of the frequently occurring sleeping disturbances in these patients. However, regardless of causality, it appears that treating with GH affects sleeping quality. For example, on GH treatment in GH-deficient patients, normalized sleeping times and increased rapid eye movement (REM) sleep were observed, whereas delta-wave sleep was unchanged[29]. Also, there have been observations of a linear relationship between the amount of slow wave (stage 3 and 4) sleep and the amount of concomitant GH secretion. Moreover, during aging, slow wave sleep and GH secretion decrease exponentially and with the same chronological pattern (for review, see [146]). These authors speculate that GH secretagogues should be used to restore GH pulsatility to normalize sleeping patterns in the elderly. In summary, it appears there is a link, perhaps even causal, between GH secretion and sleep. In some aspects, the relationship GH and sleep unsurprisingly touches on other issues, such as exercise, depression, and aging.

### Exercise

Exercise leads to increased serum levels of GH and subsequent increased IGF-I levels in serum and also in the brain[135,137]. Exercise has been shown to increase neurogenesis[128,129]. Indeed, circulating IGF-I has been shown to mediate such an effect[159]. Exercise by running in a wheel enhances the number of BrdU-labeled cells in the SGZ of the dentate gyrus in the rat hippocampus. This effect is probably not due to environmental stimulation as that process per se mainly affects survival of newborn cells and not proliferation[88,121]. Exercise is associated with a sensation of well being and this subjective state has been objectively quantified with psychometric, cardiovascular, and neurophysiological data. Cortisol and beta-endorphin levels increase transiently after exercise although the cortisol level has been shown to be below baseline hours after the exercise, which may indicate a lower stress level[76]. Correspondingly, blocking opioid receptors during exercise partly decreases the positive effect of exercise on hippocampal neurogenesis[198]. Exercise alone is also known to increase angiogenic activity in the adult rat cerebellum and hippocampus[46,196]. It may be that exercise, via running, influences neurogenesis by a common source of a vascular intermediate (stimulating both neurogenesis and angiogenesis), where the initial proliferative response in the SGZ could be caused by exercise-elevated peripheral factors, e.g., vascular endothelial growth factor-A (VEGF-A) or
IGF-I[71,116,132,135]. Another factor, in addition to IGF-I[159], that increases in the hippocampus after exercise is fibroblast growth factor-2 (FGF-2), which is a well-known mitogen of adult neural progenitors[99]. Importantly, exercise has been shown to elevate markedly both serum bFGF[137] and local hippocampal bFGF[99]. There are important interactions between IGF-I and FGF-2, as FGF-2 has been shown to increase IGF-I receptors and IGF-binding proteins[36,48], and IGF-I *in vitro* has been shown to potentiate the effect of FGF-2 on progenitor proliferation[171]. The effect of exercise (causing an increase in circulating IGF-I) on learning appears to be consistent with a transgenic model of Alzheimer's disease. Five months of voluntary exercise resulted in a decrease in extracellular amyloid-beta (A-beta) plaques in the frontal cortex and the hippocampus, as well as in an enhancement in the rate of learning in the Morris water maze, with significant reductions in escape latencies over the first trial days[206]. Interestingly, recent data indicate that the decrease in A-beta may be mediated by IGF-I via the endocytic receptor megalin/low-density lipoprotein receptor-related protein-2 (LRP2), a multicargo transporter known to participate in brain uptake of A-beta carriers[210]. It appears from these experiments that megalin mediates both IGF-I-induced clearance of A-beta, as well as being involved in IGF-I transport into the brain.

Altogether, exercise affects the CNS via multiple mechanisms. Both serum IGF-I and possibly local IGF-I appears to be involved in these processes, while GH has only been studied indirectly via IGF-I. The effects of exercise on the CNS via the elevation of IGF-I has some (but not all) features in common to the effects of GH and IGF-I on learning and memory, which is the subject of the next section.

### LEARNING AND MEMORY

GH and IGF-I treatment improves cognitive function in several ways, as shown both in animal experiments and in humans. It appears that GH improves self-reported well being[66], as well as short- and long-term memory in GH-deficient young adults[97], and probably in elderly people (for review, see [147]). Likely, part of the effect is mediated by IGF-I, as indicated by a recent study of an elderly group of humans. A high-serum IGF-I was associated with a significant increase in cerebral blood flow in the left premotor cortex during a working memory test (for easier items) and in left dorsolateral prefrontal cortex (for more difficult items), as measured by positron emission tomography[207]. Similarly, both GH[70,73] and IGF-I[101] improve performance in different tests for either short- or long-term memory in rodents. Also, it may be that learning per se enhances *local* brain IGF-I, such as has been shown for BDNF (for review, see [188]). However, to our knowledge, it is unknown whether *locally produced* GH or IGF-I are increased in the hippocampus after learning.

#### Demand of Memory Capacity — A Neuroendocrinological View

Could there be a physiological interplay between exercise and learning? Why or how would exercise, likely via circulating IGF-I, induce plastic events in the hippocampus? One explanation could be adaptations to the seasonal variation in access to food. During periods when it is difficult to find food, rodents have to run longer distances to find food and return to the nest, both requiring enhanced memory imprinting of their paths. As a result, when rodents increase their territory size for seeking food, they need enhanced spatial memory. The link to increased physical activity would increase GH and IGF-I levels, and thereby cell genesis and other aspects of plasticity and spatial memory. This assumption is supported by a study of wild meadow voles, where females, captured during the nonbreeding season while seeking food, had higher rates of cell genesis in the GCL and hilus of the dentate gyrus than did those captured during the breeding season[111]. In contrast to females, the male meadow voles increase their territory size during the breeding season. While the GCL was unaffected in cellular proliferation during the breeding season, the hilus region exhibited a great increase[111]. The authors evidenced associations to serum estradiol and testesterone levels in females and males, respectively, but we would like to speculate that GH/IGF-I was also mediating these effects, possibly secondary to a gonadal hormone concentration change. In addition, estradiol synergizes the effect of IGF-I on increasing cell genesis in the hippocampus, whereas it appears to have no effect on its own[185]. Barnea and Nottebohm also found a seasonal change in the number of neurons produced in the hippocampus of canaries that was related to territory size[60]. Thus, it is likely that increased exercise in rodents during food seeking would enhance circulating IGF-I that reaches the hippocampus[159] and subsequently increases neurogenesis and learning and spatial navigation in a dynamic manner. It is tempting to speculate that the corresponding phenomena may be active in the primate and human brain as well. A human correlate to a demand of increased spatial navigation may partly be discerned in a study that was presented a few years ago of London taxi drivers. The MRI scans of the taxi drivers showed that the posterior hippocampus, which is believed to store a spatial representation of the environment, was significantly larger and correlated to the number of years of service of the taxi drivers[138]. It appears that adult cell genesis and other events of brain plasticity would account for these macrostructural trophic increases in hippocampal volume.

### SUMMARY AND CONCLUSION

Peripheral circulating or exogenous administration of IGF-I to the adult brain affects both proliferation, cellular differentiation, and various plasticity-related processes in several regions of the brain. Importantly, IGF-I enhances adult neurogenesis in the hippocampus and increases oligodendrocyte recruitment of newborn cells in the hippocampus. The neuroprotective role of IGF-I has been studied for a number of years and is now established. The findings summarized in this paper extend the role of IGF-I as a putative regenerative agent in the adult CNS. In addition, other important mechanisms by which GH and IGF-I act on the brain are via several neurotransmitter systems, via astrocyte intercellular communication, via glucose energy metabolism, neuronal dendritic ultrastructure, and cerebral blood flow. These processes seem to be involved in various functional aspects of brain function, such as aging, stress, exercise, and learning.

GH and IGF-I have many effects in common, but there are also data suggesting some differences. Perhaps these differences will prove to be more significant in short-term treatments, since at that point secondary elevations in IGF-I would still not have come into effect.

The different mechanisms of action of GH and IGF-I, the discrimination of effects between GH and IGF-I, and whether local or systemic GH and IGF-I dominates are important areas of research; however, perhaps even more important is whether these substances can be implemented in the treatment of various diseases. For clinical purposes, GH is today mostly used in substitution treatments in patients with various kinds of insufficiency. IGF-I has not previously been tried in these situations, probably because of adverse effects (e.g. hypoglycemia) in animal experiments. From a theoretical point of view, these obstacles should be mostly a question of suitable administration, either by an appropriate systemic administration or even by supplying IGF-I intrathecally. Intrathecal administration of IGF-I or GH could be an alternative in intensive care situations where neuroprotection would be desirable. In these respects, it will be exciting to see if these agents can be used in clinical trials as treatment for either neuroprotection or in the regenerative process after human brain injury.

### ACKNOWLEDGMENTS

This study was supported by grants from the Faculty of Medicine of the University of Göteborg, the Swedish Society of Medicine, Göteborgs Läkaresällskap, and the Novo Nordisk Foundation. We thank Dr. Charles Taft (Göteborg, Sweden) for careful correction of the English manuscript.

## References

1. Schaper, A. (1897) Die fruhesten differenzierungsvorgange im centralnervensystems. *Arch. fEntw-Mech. Organ.* **5**, 81–132.
2. Kershman, J. (1938) The medulloblast and the medulloblastoma. *Arch. Neurol. Psychiatry* **40**, 937–967.
3. Hyden, H. (1962) The neuron and its glia-a biochemical and functional unit. *Endeavour* **21**, 144–155.
4. Altman, J. and Das, G.D. (1965) Autoradiographic and histological evidence of postnatal hippocampal neurogenesis in rats. *J. Comp. Neurol.* **124**, 319–336.
5. Diamond, M.C. (1968) The effects of early hypophysectomy and hormone therapy on brain development. *Brain Res.* **7**, 407–418.
6. Mayberry, H.E., Van den Brande, J.L., Van Wyk, J.J., and Waddell, W.J. (1971) Early localization of 125-I-labelled human growth hormone in adrenals and other organs of immature hypophysectomized rats. *Endocrinology* **88**, 1309–1317.
7. Stern, W.C., Miller, M., Jalowiec, J.E., Forbes, W.B., and Morgane, P.J. (1975) Effects of growth hormone on brain biogenic amine levels. *Pharmacol. Biochem. Behav.* **3**, 1115–1118.
8. Pelton, E.W., Grindeland, R.E., Young, E., and Bass, N.H. (1977) Effects of immunologically induced growth hormone deficiency on myelinogenesis in developing rat cerebrum. *Neurology* **27**, 282–288.
9. Kuhn, C.M., Butler, S.R., and Schanberg, S.M. (1978) Selective depression of serum growth hormone during maternal deprivation in rat pups. *Science* **201**, 1034–1036.
10. Pacold, S.T., Kirsteins, L., Hojvat, S., and Lawrence, A.M. (1978) Biologically active pituitary hormones in the rat brain amygdaloid nucleus. *Science* **199**, 804–806.

11. Loewenstein, W.R. (1981) Junctional intercellular communication: the cell-to-cell membrane channel. *Physiol. Rev.* **61**, 829–913.
12. Belchetz, P.E., Ridley, R.M., and Baker, H.F. (1982) Studies on the accessibility of prolactin and growth hormone to brain: effect of opiate agonists on hormone levels in serial, simultaneous plasma and cerebrospinal fluid samples in the rhesus monkey. *Brain Res.* **239**, 310–314.
13. Hojvat, S., Baker, G., Kirsteins, L., and Lawrence, A.M. (1982) Growth hormone (GH) immunoreactivity in the rodent and primate CNS: distribution, characterization and presence posthypophysectomy. *Brain Res.* **239**, 543–557.
14. Andersson, K., Fuxe, K., Eneroth, P., Isaksson, O., Nyberg, F., and Roos, P. (1983) Rat growth hormone and hypothalamic catecholamine nerve terminal systems. Evidence for rapid and discrete reductions in dopamine and noradrenaline levels and turnover in the median eminence of the hypophysectomized male rat. *Eur. J. Pharmacol.* **95**, 271–275.
15. Fischer, G. and Kettenmann, H. (1985) Cultured astrocytes form a syncytium after maturation. *Exp. Cell Res.* **159**, 273–279.
16. Hansson, E., Eriksson, P., and Nilsson, M. (1985) Amino acid and monoamine transport in primary astroglial cultures from defined brain regions. *Neurochem. Res.* **10**, 1335–1341.
17. Noguchi, T. and Sugisaki, T. (1985) Abnormal neuronal growth in the little (lit) cerebrum. *Exp. Neurol.* **89**, 274–278.
18. Camel, J.E., Withers, G.S., and Greenough, W.T. (1986) Persistence of visual cortex dendritic alterations induced by postweaning exposure to a "superenriched" environment in rats. *Behav. Neurosci.* **100**, 810–813.
19. Mathews, L.S., Norstedt, G., and Palmiter, R.D. (1986) Regulation of insulin-like growth factor I gene expression by growth hormone. *Proc. Natl. Acad. Sci. U. S. A.* **83**, 9343–9347.
20. Gossard, F., Dihl, F., Pelletier, G., Dubois, P.M., and Morel, G. (1987) In situ hybridization to rat brain and pituitary gland of growth hormone cDNA. *Neurosci. Lett.* **79**, 251–256.
21. Linkowski, P., Mendlewicz, J., Kerkhofs, M., Leclercq, R., Golstein, J., Brasseur, M., Copinschi, G., and Van Cauter, E. (1987) 24-hour profiles of adrenocorticotropin, cortisol, and growth hormone in major depressive illness: effect of antidepressant treatment. *J. Clin. Endocrinol. Metab.* **65**, 141–152.
22. DiCicco Bloom, E. and Black, I.B. (1988) Insulin growth factors regulate the mitotic cycle in cultured rat sympathetic neuroblasts. *Proc. Natl. Acad. Sci. U. S. A.* **85**, 4066–4070.
23. Duffy, K.R., Pardridge, W.M., and Rosenfeld, R.G. (1988) Human blood-brain barrier insulin-like growth factor receptor. *Metabolism* **37**, 136–140.
24. Lesniak, M.A., Hill, J.M., Kiess, W., Rojeski, M., Pert, C.B., and Roth, J. (1988) Receptors for insulin-like growth factors I and II: autoradiographic localization in rat brain and comparison to receptors for insulin. *Endocrinology* **123**, 2089–2099.
25. Araujo, D.M., Lapchak, P.A., Collier, B., Chabot, J.G., and Quirion, R. (1989) Insulin-like growth factor-1 (somatomedin-C) receptors in the rat brain: distribution and interaction with the hippocampal cholinergic system. *Brain Res.* **484**, 130–138.
26. Bryan, G.K. and Riesen, A.H. (1989) Deprived somatosensory-motor experience in stumptailed monkey neocortex: dendritic spine density and dendritic branching of layer IIIB pyramidal cells [published erratum appears in *J. Comp. Neurol*. (1989) **289(4),** 709]. *J. Comp. Neurol.* **286**, 208–217.
27. Daughaday, W.H. and Rotwein, P. (1989) Insulin-like growth factors I and II. Peptide, messenger ribonucleic acid and gene structures, serum, and tissue concentrations. *Endocr. Rev.* **10**, 68–91.
28. Morisawa, K., Sugisaki, T., Kanamatsu, T., Aoki, T., and Noguchi, T. (1989) Factors contributing to cerebral hypomyelination in the growth hormone-deficient little mouse. *Neurochem. Res.* **14**, 173–177.
29. Åström, C., Pedersen, S.A., and Lindholm, J. (1990) The influence of growth hormone on sleep in adults with growth hormone deficiency. *Clin. Endocrinol. Oxf.* **33**, 495–500.
30. Hansson, E. and Rönnbäck, L. (1990) Astrocytes in neurotransmission. A review. *Cell. Mol. Biol.* **36,** 487–496.
31. Sara, V.R. and Hall, K. (1990) Insulin-like growth factors and their binding proteins. *Physiol. Rev.* **70**, 591–614.
32. Werther, G.A., Abate, M., Hogg, A., Cheesman, H., Oldfield, B., Hards, D., Hudson, P., Power, B., Freed, K., and Herington, A.C. (1990) Localization of insulin-like growth factor-I mRNA in rat brain by in situ hybridization — relationship to IGF-I receptors. *Mol. Endocrinol.* **4**, 773–778.
33. Arce, V.M., Cella, S.G., Locatelli, V., and Müller, E.E. (1991) Studies of growth hormone secretion in calorically restricted dogs: effect of cholinergic agonists and antagonists, glucose and thyrotropin-releasing hormone. *Neuroendocrinology* **53**, 467–472.
34. Ayer le Lievre, C., Stahlbom, P.A., and Sara, V.R. (1991) Expression of IGF-I and -II mRNA in the brain and craniofacial region of the rat fetus. *Development* **111**, 105–115.
35. Bach, M.A., Shen Orr, Z., Lowe, Jr., W.L., Roberts, Jr., C.T., and LeRoith, D. (1991) Insulin-like growth factor I mRNA levels are developmentally regulated in specific regions of the rat brain. *Brain Res. Mol. Brain Res.* **10**, 43–48.
36. Drago, J., Murphy, M., Carroll, S.M., Harvey, R.P., and Bartlett, P.F. (1991) Fibroblast growth factor-mediated proliferation of central nervous system precursors depends on endogenous production of insulin-like growth factor I. *Proc. Natl. Acad. Sci. U. S. A.* **88**, 2199–2203.
37. Lai, Z.N., Emtner, M., Roos, P., and Nyberg, F. (1991) Characterization of putative growth hormone receptors in human choroid plexus. *Brain Res.* **546**, 222–226.
38. Marks, J.L., Porte, Jr., D., and Baskin, D.G. (1991) Localization of type I insulin-like growth factor receptor messenger RNA in the adult rat brain by in situ hybridization. *Mol. Endocrinol.* **5**, 1158–1168.

39. Martinoli, M.G., Ouellet, J., Rheaume, E., and Pelletier, G. (1991) Growth hormone and somatostatin gene expression in adult and aging rats as measured by quantitative in situ hybridization. *Neuroendocrinology* **54**, 607–615.
40. Masters, B.A., Werner, H., Roberts, Jr., C.T., LeRoith, D., and Raizada, M.K. (1991) Developmental regulation of insulin-like growth factor-I-stimulated glucose transporter in rat brain astrocytes. *Endocrinology* **128**, 2548–2557.
41. Bartlett, W.P., Li, X.S., and Williams, M. (1992) Expression of IGF-1 mRNA in the murine subventricular zone during postnatal development. *Brain Res. Mol. Brain Res.* **12**, 285–291.
42. Bondy, C., Werner, H., Roberts, Jr., C.T., and LeRoith, D. (1992) Cellular pattern of type-I insulin-like growth factor receptor gene expression during maturation of the rat brain: comparison with insulin-like growth factors I and II. *Neuroscience* **46**, 909–923.
43. Burton, K.A., Kabigting, E.B., Clifton, D.K., and Steiner, R.A. (1992) Growth hormone receptor messenger ribonucleic acid distribution in the adult male rat brain and its colocalization in hypothalamic somatostatin neurons. *Endocrinology* **131**, 958–963.
44. Ghigo, E., Goffi, S., Arvat, E., Imperiale, E., Boffano, G.M., Valetto, M.R., Mazza, E., Santi, I., Magliona, A., and Boghen, M.F. (1992) A neuroendocrinological approach to evidence an impairment of central cholinergic function in aging. *J. Endocrinol. Invest.* **15**, 665–670.
45. Gluckman, P., Klempt, N., Guan, J., Mallard, C., Sirimanne, E., Dragunow, M., Klempt, M., Singh, K., Williams, C., and Nikolics, K. (1992) A role for IGF-1 in the rescue of CNS neurons following hypoxic-ischemic injury. *Biochem. Biophys. Res. Commun.* **18**2, 593–599.
46. Isaacs, K.R., Anderson, B.J., Alcantara, A.A., Black, J.E., and Greenough, W.T. (1992) Exercise and the brain: angiogenesis in the adult rat cerebellum after vigorous physical activity and motor skill learning [published erratum appears in *J. Cereb. Blood Flow Metab.* (1992) **12(3)**, 533]. *J. Cereb. Blood Flow Metab.* **12,** 110–119.
47. Lobie, P.E., Garcia Aragon, J., Wang, B.S., Baumbach, W.R., and Waters, M.J. (1992) Cellular localization of the growth hormone binding protein in the rat. *Endocrinology* **130**, 3057–3065.
48. Pons, S. and Torres Aleman, I. (1992) Basic fibroblast growth factor modulates insulin-like growth factor-I, its receptor, and its binding proteins in hypothalamic cell cultures. *Endocrinology* **131**, 2271–2278.
49. Reynolds, B.A. and Weiss, S. (1992) Generation of neurons and astrocytes from isolated cells of the adult mammalian central nervous system. *Science* **255**, 1707–1710.
50. Richards, L.J., Kilpatrick, T.J., and Bartlett, P.F. (1992) De novo generation of neuronal cells from the adult mouse brain. *Proc. Natl. Acad. Sci. U. S. A.* **89**, 8591–8595.
51. Aguado, F., Rodrigo, J., Cacicedo, L., and Mellström, B. (1993) Distribution of insulin-like growth factor-I receptor mRNA in rat brain. Regulation in the hypothalamo-neurohypophysial system. *J. Mol. Endocrinol.* **11**, 231–239.
52. Beilharz, E.J., Klempt, N.D., Klempt, M., Sirimanne, E., Dragunow, M., and Gluckman, P.D. (1993) Differential expression of insulin-like growth factor binding proteins (IGFBP) 4 and 5 mRNA in the rat brain after transient hypoxic-ischemic injury. *Brain Res. Mol. Brain Res.* **18,** 209–215.
53. Bondy, C. and Lee, W.H. (1993) Correlation between insulin-like growth factor (IGF)-binding protein 5 and IGF-I gene expression during brain development. *J. Neurosci.* **13**, 5092–5104.
54. Bozyczko Coyne, D., Glicksman, M.A., Prantner, J.E., McKenna, B., Connors, T., Friedman, C., Dasgupta, M., and Neff, N.T. (1993) IGF-I supports the survival and/or differentiation of multiple types of central nervous system neurons. *Ann. N. Y. Acad. Sci.* **692**, 311–313.
55. Carson, M.J., Behringer, R.R., Brinster, R.L., and McMorris, F.A. (1993) Insulin-like growth factor I increases brain growth and central nervous system myelination in transgenic mice. *Neuron* **10**, 729–740.
56. Guan, J., Williams, C., Gunning, M., Mallard, C., and Gluckman, P. (1993) The effects of IGF-1 treatment after hypoxic-ischemic brain injury in adult rats. *J. Cereb. Blood Flow Metab.* **13,** 609–616.
57. Lee, W.H., Michels, K.M., and Bondy, C.A. (1993) Localization of insulin-like growth factor binding protein-2 messenger RNA during postnatal brain development: correlation with insulin-like growth factors I and II. *Neuroscience* **53**, 251–265.
58. Lobie, P.E., Garcia Aragon, J., Lincoln, D.T., Barnard, R., Wilcox, J.N., and Waters, M.J. (1993) Localization and ontogeny of growth hormone receptor gene expression in the central nervous system. *Brain Res. Dev. Brain Res.* **74**, 225–233.
59. Nilsson-Håkansson, L., Civalero, I., Zhang, X., Carlsson-Skwirut, C., Sara, V.R., and Nordberg, A. (1993) Effects of IGF-1, truncated IGF-1 and the tripeptide Gly-Pro-Glu on acetylcholine release from parietal cortex of rat brain. *Neuroreport* **4**, 1111–1114.
60. Barnea, A. and Nottebohm, F. (1994) Seasonal recruitment of hippocampal neurons in adult free-ranging black-capped chickadees. *Proc. Natl. Acad. Sci. U. S. A.* **91**, 11217–11221.
61. Duenas, M., Luquin, S., Chowen, J.A., Torres Aleman, I., Naftolin, F., and Garcia Segura, L.M. (1994) Gonadal hormone regulation of insulin-like growth factor-I-like immunoreactivity in hypothalamic astroglia of developing and adult rats. *Neuroendocrinology* **59**, 528–538.
62. Logan, A., Gonzalez, A.M., Hill, D.J., Berry, M., Gregson, N.A., and Baird, A. (1994) Coordinated pattern of expression and localization of insulin-like growth factor-II (IGF-II) and IGF-binding protein-2 in the adult rat brain. *Endocrinology* **135**, 2255–2264.
63. Russo, V.C. and Werther, G.A. (1994) Des (1-3) IGF-I potently enhances differentiated cell growth in olfactory bulb organ culture. *Growth Factors* **11**, 301–311.

64. Stenvers, K.L., Zimmermann, E.M., Gallagher, M., and Lund, P.K. (1994) Expression of insulin-like growth factor binding protein-4 and -5 mRNAs in adult rat forebrain. *J. Comp. Neurol.* **339**, 91–105.
65. Beck, K.D., Powell-Braxton, L., Widmer, H.R., Valverde, J., and Hefti, F. (1995) IGF-I gene disruption results in reduced brain size, CNS hypomyelination, and loss of hippocampal granule and striatal parvalbumin-containing neurons. *Neuron* **14**, 717–730.
66. Burman, P., Broman, J.E., Hetta, J., Wiklund, I., Erfurth, E.M., Hagg, E., and Karlsson, F.A. (1995) Quality of life in adults with growth hormone (GH) deficiency: response to treatment with recombinant human GH in a placebo-controlled 21-month trial. *J. Clin. Endocrinol. Metab.* **80**, 3585–3590.
67. Huard, J.M. and Schwob, J.E. (1995) Cell cycle of globose basal cells in rat olfactory epithelium. *Dev. Dyn.* **203**, 17–26.
68. Johansson, J.-O., Larson, G., Andersson, M., Elmgren, A., Hynsjö, L., Lindahl, A., Lundberg, P.-A., Isaksson, O.G.P., Lindstedt, S., and Bengtsson, B.-Å. (1995) Treatment of growth hormone-deficient adults with recombinant human growth hormone increases the concentration of growth hormone in the cerebrospinal fluid and affects neurotransmitters. *Neuroendocrinology* **61**, 57–66.
69. Miller, D.B., Bartke, A., and O'Callaghan, J.P. (1995) Increased glial fibrillary acidic protein (GFAP) levels in the brains of transgenic mice expressing the bovine growth hormone (bGH) gene. *Exp. Gerontol*. **30**, 383–400.
70. Schneider Rivas, S., Rivas Arancibia, S., Vazquez Pereyra, F., Vazquez Sandoval, R., and Borgonio Perez, G. (1995) Modulation of long-term memory and extinction responses induced by growth hormone (GH) and growth hormone releasing hormone (GHRH) in rats. *Life Sci.* **56**, PL433–PL441.
71. Breen, E.C., Johnson, E.C., Wagner, H., Tseng, H.M., Sung, L.A., and Wagner, P.D. (1996) Angiogenic growth factor mRNA responses in muscle to a single bout of exercise. *J. Appl. Physiol.* **81**, 355–361.
72. D'Ercole, A.J., Ye, P., Calikoglu, A.S., and Gutierrez Ospina, G. (1996) The role of the insulin-like growth factors in the central nervous system. *Mol. Neurobiol.* **13**, 227–255.
73. Drago, F., Dileo, F., Ikonomou, S., Anzallo, C., Busa, L., and Lopresti, L. (1996) Behavioral and endocrine effects of growth hormone administration in aged female rats. *Psychoneuroendocrinology* **21**, 401–410.
74. Flavell, D.M., Wells, T., Wells, S.E., Carmignac, D.F., Thomas, G.B., and Robinson, I.C. (1996) Dominant dwarfism in transgenic rats by targeting human growth hormone (GH) expression to hypothalamic GH-releasing factor neurons. *EMBO J.* **15**, 3871–3879.
75. Guan, J., Skinner, S.J., Beilharz, E.J., Hua, K.M., Hodgkinson, S., Gluckman, P.D., and Williams, C.E. (1996) The movement of IGF-I into the brain parenchyma after hypoxic-ischaemic injury. *Neuroreport* **7**, 632–636.
76. Heitkamp, H.C., Huber, W., and Scheib, K. (1996) beta-Endorphin and adrenocorticotrophin after incremental exercise and marathon running — female responses. *Eur. J. Appl. Physiol. Occup. Physiol.* **72,** 417–424.
77. Johnston, B.M., Mallard, E.C., Williams, C.E., and Gluckman, P.D. (1996) Insulin-like growth factor-1 is a potent neuronal rescue agent after hypoxic-ischemic injury in fetal lambs. *J. Clin. Invest.* **97**, 300–308.
78. Kuhn, H.G., Dickinson Anson, H., and Gage, F.H. (1996) Neurogenesis in the dentate gyrus of the adult rat: age-related decrease of neuronal progenitor proliferation. *J. Neurosci.* **16**, 2027–2033.
79. Lee, W.H., Wang, G.M., Seaman, L.B., and Vannucci, S.J. (1996) Coordinate IGF-I and IGFBP5 gene expression in perinatal rat brain after hypoxia-ischemia. *J. Cereb. Blood Flow Metab.* **16**, 227–236.
80. Lopezfernandez, J., Sanchezfranco, F., Velasco, B., Tolon, R.M., Pazos, F., and Cacicedo, L. (1996) Growth hormone induces somatostatin and insulin like growth factor I gene expression in the cerebral hemispheres of aging rats. *Endocrinology* **137**, 4384–4391.
81. Tabernero, A., Giaume, C., and Medina, J.M. (1996) Endothelin-1 regulates glucose utilization in cultured astrocytes by controlling intercellular communication through gap junctions. *Glia* **16,** 187–195.
82. Ye, P., Xing, Y., Dai, Z., and D'Ercole, A.J. (1996) In vivo actions of insulin-like growth factor-I (IGF-I) on cerebellum development in transgenic mice: evidence that IGF-I increases proliferation of granule cell progenitors. *Brain Res. Dev. Brain Res.* **95**, 44–54.
83. Chen, L., Lund, P.K., Burgess, S.B., Rudisch, B.E., and McIlwain, D.L. (1997) Growth hormone, insulin-like growth factor I, and motoneuron size. *J. Neurobiol.* **32,** 202–212.
84. Feldman, E.L., Sullivan, K.A., Kim, B., and Russell, J.W. (1997) Insulin-like growth factors regulate neuronal differentiation and survival. *Neurobiol. Dis.* **4**, 201–214.
85. Garcia-Segura, L.M., Rodriguez, J.R., and Torres-Aleman, I. (1997) Localization of the insulin-like growth factor I receptor in the cerebellum and hypothalamus of adult rats: an electron microscopic study. *J. Neurocytol.* **26**, 479–490.
86. Giaume, C., Tabernero, A., and Medina, J.M. (1997) Metabolic trafficking through astrocytic gap junctions. *Glia* **21**, 114–123.
87. Gillespie, C.M., Merkel, A.L., and Martin, A.A. (1997) Effects of insulin-like growth factor-I and LR3IGF-I on regional blood flow in normal rats. *J. Endocrinol.* **155**, 351–358.
88. Kempermann, G., Kuhn, H.G., and Gage, F.H. (1997) More hippocampal neurons in adult mice living in an enriched environment. *Nature* **386**, 493–495.
89. Nieto Bona, M.P., Garcia Segura, L.M., and Torres Aleman, I. (1997) Transynaptic modulation by insulin-like growth factor I of dendritic spines in Purkinje cells. *Int. J. Dev. Neurosci.* **15**, 749–754.
90. Parrizas, M., Saltiel, A.R., and LeRoith, D. (1997) Insulin-like growth factor 1 inhibits apoptosis using the phosphatidylinositol 3'-kinase and mitogen-activated protein kinase pathways. *J. Biol. Chem.* **272**, 154–161.
91. Schaeffer, H.J. and Sirotkin, A.V. (1997) Melatonin and serotonin regulate the release of insulin-like growth factor-I, oxytocin and progesterone by cultured human granulosa cells. *Exp. Clin. Endocrinol. Diabetes* **105**, 109–112.

92. Sonntag, W.E., Lynch, C.D., Cooney, P.T., and Hutchins, P.M. (1997) Decreases in cerebral microvasculature with age are associated with the decline in growth hormone and insulin-like growth factor 1. *Endocrinology* **138**, 3515–3520.
93. Tagami, M., Ikeda, K., Nara, Y., Fujino, H., Kubota, A., Numano, F., and Yamori, Y. (1997) Insulin-like growth factor-1 attenuates apoptosis in hippocampal neurons caused by cerebral ischemia and reperfusion in stroke-prone spontaneously hypertensive rats. *Lab. Invest.* **76,** 613–617.
94. Wiesinger, H., Hamprecht, B., and Dringen, R. (1997) Metabolic pathways for glucose in astrocytes. *Glia* **21**, 22–34.
95. Beilharz, E.J., Russo, V.C., Butler, G., Baker, N.L., Connor, B., Sirimanne, E.S., Dragunow, M., Werther, G.A., Gluckman, P.D., Williams, C.E., and Scheepens, A. (1998) Co-ordinated and cellular specific induction of the components of the IGF/IGFBP axis in the rat brain following hypoxic-ischemic injury. *Brain Res. Mol. Brain Res.* **59**, 119–134.
96. Cheng, C.M., Joncas, G., Reinhardt, R.R., Farrer, R., Quarles, R., and Janssen, J. (1998) Biochemical and morphometric analyses show that myelination in the insulin-like growth factor 1 null brain is proportionate to its neuronal composition. *J. Neurosci.* **18**, 5673–5681.
97. Deijen, J.B., de Boer, H., and van der Veen, E.A. (1998) Cognitive changes during growth hormone replacement in adult men. *Psychoneuroendocrinology* **23**, 45–55.
98. Eriksson, P.S., Perfilieva, E., Björk-Eriksson, T., Alborn, A.-M., Nordborg, C., Peterson, D.A., and Gage, F.H. (1998) Neurogenesis in the adult human hippocampus. *Nat. Med.* **4**, 1313–1317.
99. Gomez-Pinilla, F., So, V., and Kesslak, J.P. (1998) Spatial learning and physical activity contribute to the induction of fibroblast growth factor: neural substrates for increased cognition associated with exercise. *Neuroscience* **85**, 53–61.
100. Kempermann, G., Kuhn, H.G., and Gage, F.H. (1998) Experience-induced neurogenesis in the senescent dentate gyrus. *J. Neurosci.* **18**, 3206–3212.
101. Markowska, A.L., Mooney, M., and Sonntag, W.E. (1998) Insulin-like growth factor-1 ameliorates age-related behavioral deficits. *Neuroscience* **87**, 559–569.
102. Russell, J.W., Windebank, A.J., Schenone, A., and Feldman, E.L. (1998) Insulin-like growth factor-I prevents apoptosis in neurons after nerve growth factor withdrawal. *J. Neurobiol.* **36,** 455–467.
103. Sakkas, P.N., Soldatos, C.R., Bergiannaki, J.D., Paparrigopoulos, T.J., and Stefanis, C.N. (1998) Growth hormone secretion during sleep in male depressed patients. *Prog. Neuropsychopharmacol. Biol. Psychiatry* **3**, 467–483.
104. Werther, G.A., Russo, V., Baker, N., and Butler, G. (1998) The role of the insulin-like growth factor system in the developing brain. *Horm. Res.* **49(Suppl 1),** 37–40.
105. Brezun, J.M. and Daszuta, A. (1999) Depletion in serotonin decreases neurogenesis in the dentate gyrus and the subventricular zone of adult rats. *Neuroscience* **89**, 999–1002.
106. Chiasson, B.J., Tropepe, V., Morshead, C.M., and van der Kooy, D. (1999) Adult mammalian forebrain ependymal and subependymal cells demonstrate proliferative potential, but only subependymal cells have neural stem cell characteristics[in process citation]. *J. Neurosci.* **19,** 4462–4471.
107. Delaney, C.L., Cheng, H.L., and Feldman, E.L. (1999) Insulin-like growth factor-I prevents caspase-mediated apoptosis in Schwann cells. *J. Neurobiol.* **41**, 540–548.
108. Dentremont, K.D., Ye, P., D'Ercole, A.J., and O'Kusky, J.R. (1999) Increased insulin-like growth factor-I (IGF-I) expression during early postnatal development differentially increases neuron number and growth in medullary nuclei of the mouse. *Brain Res. Dev. Brain Res.* **114**, 135–141.
109. Doetsch, F., Caille, I., Lim, D.A., Garcia-Verdugo, J.M., and Alvarez-Buylla, A. (1999) Subventricular zone astrocytes are neural stem cells in the adult mammalian brain. *Cell* **97**, 703–716.
110. Fernandez, A.M., De La Vega, A.G., Planas, B., and Torres-Aleman, I. (1999) Neuroprotective actions of peripherally administered insulin-like growth factor I in the injured olivo-cerebellar pathway. *Eur. J. Neurosci.* **11**, 2019–2030.
111. Galea, L.A. and McEwen, B.S. (1999) Sex and seasonal differences in the rate of cell proliferation in the dentate gyrus of adult wild meadow voles. *Neuroscience* **89**, 955–964.
112. Gould, E. (1999) Serotonin and hippocampal neurogenesis. *Neuropsychopharmacology* **21,** 46S–51S.
113. Gould, E., Beylin, A., Tanapat, P., Reeves, A., and Shors, T.J. (1999) Learning enhances adult neurogenesis in the hippocampal formation[see comments]. *Nat. Neurosci.* **2**, 260–265.
114. Gould, E., Reeves, A.J., Graziano, M.S., and Gross, C.G. (1999) Neurogenesis in the neocortex of adult primates. *Science* **286**, 548–552.
115. Gustafson, K., Hagberg, H., Bengtsson, B.A., Brantsing, C., and Isgaard, J. (1999) Possible protective role of growth hormone in hypoxia-ischemia in neonatal rats. *Pediatr. Res.* **45**, 318–323.
116. Gustafsson, T., Puntschart, A., Kaijser, L., Jansson, E., and Sundberg, C.J. (1999) Exercise-induced expression of angiogenesis-related transcription and growth factors in human skeletal muscle. *Am. J. Physiol.* **276**, H679–H685.
117. Johansson, C.B., Momma, S., Clarke, D.L., Risling, M., Lendahl, U., and Frisén, J. (1999) Identification of a neural stem cell in the adult mammalian central nervous system. *Cell* **96**, 25–34.
118. Lambert, H.W. and Lauder, J.M. (1999) Serotonin receptor agonists that increase cyclic AMP positively regulate IGF-I in mouse mandibular mesenchymal cells. *Dev. Neurosci.* **21**, 105–112.
119. Leventhal, P.S., Russel, J.W., and Feldman, E.L. (1999) IGF's and the nervous system. In *The IGF System*. Rosenfeldt, R.G. and Roberts, C.T., Eds. Humana Press, Portland, OR. pp. 425–455.
120. Levison, S.W., Young, G.M., and Goldman, J.E. (1999) Cycling cells in the adult rat neocortex preferentially generate oligodendroglia. *J. Neurosci. Res.* **57**, 435–446.

121. Nilsson, M., Perfilieva, E., Johansson, U., Orwar, O., and Eriksson, P.S. (1999) Enriched environment increases neurogenesis in the adult rat dentate gyrus and improves spatial memory. *J. Neurobiol.* **39**, 569–578.
122. Oscarsson, J., Ottosson, M., Vikman-Adolfsson, K., Frick, F., Enerbäck, S., Lithell, H., and Eden, S. (1999) GH but not IGF-I or insulin increases lipoprotein lipase activity in muscle tissues of hypophysectomised rats. *J. Endocrinol.* **160**, 247–255.
123. Peruzzi, F., Prisco, M., Dews, M., Salomoni, P., Grassilli, E., Romano, G., Calabretta, B., and Baserga, R. (1999) Multiple signaling pathways of the insulin-like growth factor 1 receptor in protection from apoptosis. *Mol. Cell. Biol.* **19**, 7203–7215.
124. Scheepens, A., Sirimanne, E., Beilharz, E., Breier, B.H., Waters, M.J., Gluckman, P.D., and Williams, C.E. (1999) Alterations in the neural growth hormone axis following hypoxic-ischemic brain injury. *Brain Res. Mol. Brain Res.* **68**, 88–100.
125. Sjögren, K., Liu, J.L., Blad, K., Skrtic, S., Vidal, O., Wallenius, V., LeRoith, D., Törnell, J., Isaksson, O.G.P., Jansson, J.-O., and Ohlsson, C. (1999) Liver-derived insulin-like growth factor I (IGF-I) is the principal source of IGF-I in blood but is not required for postnatal body growth in mice. *Proc. Natl. Acad. Sci. U. S. A.* **96,** 7088–7092.
126. Söderpalm, B., Ericson, M., Bohlooly, M., Engel, J.A., and Tornell, J. (1999) Bovine growth hormone transgenic mice display alterations in locomotor activity and brain monoamine neurochemistry. *Endocrinology* **140**, 5619–5625.
127. Takadera, T., Matsuda, I., and Ohyashiki, T. (1999) Apoptotic cell death and caspase-3 activation induced by N-methyl-D-aspartate receptor antagonists and their prevention by insulin-like growth factor I. *J. Neurochem.* **73**, 548–556.
128. van Praag, H., Christie, B.R., Sejnowski, T.J., and Gage, F.H. (1999) Running enhances neurogenesis, learning, and long-term potentiation in mice. *Proc. Natl. Acad. Sci. U. S. A.* **96**, 13427–13431.
129. van Praag, H., Kempermann, G., and Gage, F.H. (1999) Running increases cell proliferation and neurogenesis in the adult mouse dentate gyrus[see comments]. *Nat. Neurosci.* **2**, 266–270.
130. von Werder, K. (1999) The somatopause is no indication for growth hormone therapy. *J. Endocrinol. Invest.* **22**, 137–141.
131. Walter, H.J., Berry, M., Hill, D.J., Cwyfan-Hughes, S., Holly, J.M., and Logan, A. (1999) Distinct sites of insulin-like growth factor (IGF)-II expression and localization in lesioned rat brain: possible roles of IGF binding proteins (IGFBPs) in the mediation of IGF-II activity. *Endocrinology* **140**, 520–532.
132. Åberg, M.A.I., Åberg, N.D., Hedbäcker, H., Oscarsson, J., and Eriksson, P.S. (2000) Peripheral infusion of IGF-I selectively induces neurogenesis in the adult rat hippocampus. *J. Neurosci.* **20**, 2896–2903.
133. Åberg, N.D., Carlsson, B., Rosengren, L., Oscarsson, J., Isaksson, O.G.P., Rönnbäck, L., and Eriksson, P. (2000) Growth hormone increases connexin43 expression in the cerebral cortex and hypothalamus. *Endocrinology* **141,** 3879–3886.
134. Armstrong, C.S., Wuarin, L., and Ishii, D.N. (2000) Uptake of circulating insulin-like growth factor-I into the cerebrospinal fluid of normal and diabetic rats and normalization of IGF-II mRNA content in diabetic rat brain. *J. Neurosci. Res.* **59**, 649–660.
135. Carro, E., Nunez, A., Busiguina, S., and Torres-Aleman, I. (2000) Circulating insulin-like growth factor I mediates effects of exercise on the brain. *J. Neurosci.* **20**, 2926–2933.
136. Cheng, C.M., Reinhardt, R.R., Lee, W.H., Joncas, G., Patel, S.C., and Bondy, C.A. (2000) Insulin-like growth factor 1 regulates developing brain glucose metabolism. *Proc. Natl. Acad. Sci. U. S. A.* **97**, 10236–10241.
137. Eliakim, A., Oh, Y., and Cooper, D.M. (2000) Effect of single wrist exercise on fibroblast growth factor-2, insulin-like growth factor, and growth hormone. *Am. J. Physiol. Regul. Integr. Comp. Physiol.* **279**, R548–R553.
138. Maguire, E.A., Gadian, D.G., Johnsrude, I.S., Good, C.D., Ashburner, J., Frackowiak, R.S., and Frith, C.D. (2000) Navigation-related structural change in the hippocampi of taxi drivers. *Proc. Natl. Acad. Sci. U. S. A.* **97**, 4398–4403.
139. Malberg, J.E., Eisch, A.J., Nestler, E.J., and Duman, R.S. (2000) Chronic antidepressant treatment increases neurogenesis in adult rat hippocampus. *J. Neurosci.* **20**, 9104–9110.
140. Niblock, M.M., Brunso-Bechtold, J.K., and Riddle, D.R. (2000) Insulin-like growth factor I stimulates dendritic growth in primary somatosensory cortex. *J. Neurosci.* **20**, 4165–4176.
141. O'Kusky, J.R., Ye, P., and D'Ercole, A.J. (2000) Insulin-like growth factor-I promotes neurogenesis and synaptogenesis in the hippocampal dentate gyrus during postnatal development. *J. Neurosci.* **20**, 8435–8442.
142. Roy, N.S., Wang, S., Jiang, L., Kang, J., Benraiss, A., Harrison-Restelli, C., Fraser, R.A., Couldwell, W.T., Kawaguchi, A., Okano, H., Nedergaard, M., and Goldman, S.A. (2000) In vitro neurogenesis by progenitor cells isolated from the adult human hippocampus[see comments]. *Nat. Med.* **6**, 271–277.
143. Sapolsky, R.M. (2000) Glucocorticoids and hippocampal atrophy in neuropsychiatric disorders. *Arch. Gen. Psychiatry* **57**, 925–935.
144. Shughrue, P.J. and Merchenthaler, I. (2000) Estrogen is more than just a "sex hormone": novel sites for estrogen action in the hippocampus and cerebral cortex. *Front. Neuroendocrinol.* **21**, 95–101.
145. Sonntag, W.E., Lynch, C., Thornton, P., Khan, A., Bennett, S., and Ingram, R. (2000) The effects of growth hormone and IGF-1 deficiency on cerebrovascular and brain ageing. *J. Anat.* **197(Pt 4),** 575–585.
146. Van Cauter, E. and Copinschi, G. (2000) Interrelationships between growth hormone and sleep. *Growth Hormone and IGF Research* 10 Suppl B, S57-S62.
147. van Dam, P.S., Aleman, A., de Vries, W.R., Deijen, J.B., van der Veen, E.A., de Haan, E.H. and Koppeschaar, H.P. (2000) Growth hormone, insulin-like growth factor I and cognitive function in adults. *Growth Horm. IGF Res.* **10(Suppl B),** S69– S73.

148. Van Golen, C.M. and Feldman, E.L. (2000) Insulin-like growth factor I is the key growth factor in serum that protects neuroblastoma cells from hyperosmotic-induced apoptosis. *J. Cell Physiol.* **182**, 24–32.
149. Zheng, W.H., Kar, S., Doré, S., and Quirion, R. (2000) Insulin-like growth factor-1 (IGF-1): a neuroprotective trophic factor acting via the Akt kinase pathway. *J. Neural Transm. Suppl.* **60**, 261–272.
150. Arsenijevic, Y., Weiss, S., Schneider, B., and Aebischer, P. (2001) Insulin-like growth factor-I is necessary for neural stem cell proliferation and demonstrates distinct actions of epidermal growth factor and fibroblast growth factor-2. *J. Neurosci.* **21**, 7194–7202.
151. Carro, E., Trejo, J.L., Busiguina, S., and Torres-Aleman, I. (2001) Circulating insulin-like growth factor I mediates the protective effects of physical exercise against brain insults of different etiology and anatomy. *J. Neurosci.* **21**, 5678–5684.
152. Cheng, C.M., Cohen, M., Wang, J., and Bondy, C.A. (2001) Estrogen augments glucose transporter and IGF1 expression in primate cerebral cortex. *FASEB J.* **15**, 907–915.
153. Guan, J., Bennet, L., George, S., Wu, D., Waldvogel, H.J., Gluckman, P.D., Faull, R.L., Crosier, P.S., and Gunn, A.J. (2001) Insulin-like growth factor-1 reduces postischemic white matter injury in fetal sheep. *J. Cereb. Blood Flow Metab.* **21**, 493–502.
154. Lambert, H.W., Weiss, E.R., and Lauder, J.M. (2001) Activation of 5-HT receptors that stimulate the adenylyl cyclase pathway positively regulates IGF-I in cultured craniofacial mesenchymal cells. *Dev. Neurosci.* **23**, 70–77.
155. Lichtenwalner, R.J., Forbes, M.E., Bennett, S.A., Lynch, C.D., Sonntag, W.E., and Riddle, D.R. (2001) Intracerebroventricular infusion of insulin-like growth factor-I ameliorates the age-related decline in hippocampal neurogenesis. *Neuroscience* **107**, 603–613.
156. Lynch, C.D., Lyons, D., Khan, A., Bennett, S.A., and Sonntag, W.E. (2001) Insulin-like growth factor-1 selectively increases glucose utilization in brains of aged animals. *Endocrinology* **142**, 506–509.
157. Sapolsky, R.M. (2001) Depression, antidepressants, and the shrinking hippocampus. *Proc. Natl. Acad. Sci. U. S. A.* **98**, 12320–12322.
158. Scheepens, A., Sirimanne, E.S., Breier, B.H., Clark, R.G., Gluckman, P.D., and Williams, C.E. (2001) Growth hormone as a neuronal rescue factor during recovery from CNS injury. *Neuroscience* **104**, 677–687.
159. Trejo, J.L., Carro, E., and Torres-Aleman, I. (2001) Circulating insulin-like growth factor I mediates exercise-induced increases in the number of new neurons in the adult hippocampus. *J. Neurosci.* **21**, 1628–1634.
160. Yan, H., Bunge, M.B., Wood, P.M., and Plant, G.W. (2001) Mitogenic response of adult rat olfactory ensheathing glia to four growth factors. *Glia* **33**, 334–342.
161. Bernier, P.J., Bedard, A., Vinet, J., Levesque, M., and Parent, A. (2002) Newly generated neurons in the amygdala and adjoining cortex of adult primates. *Proc. Natl. Acad. Sci. U. S. A.* **99**, 11464–11469.
162. Chung, Y.H., Shin, C.M., Joo, K.M., Kim, M.J., and Cha, C.I. (2002) Region-specific alterations in insulin-like growth factor receptor type I in the cerebral cortex and hippocampus of aged rats. *Brain Res.* **946**, 307–313.
163. Frago, L.M., Paneda, C., Dickson, S.L., Hewson, A.K., Argente, J., and Chowen, J.A. (2002) Growth hormone (GH) and GH-releasing peptide-6 increase brain insulin-like growth factor-I expression and activate intracellular signaling pathways involved in neuroprotection. *Endocrinology* **143**, 4113–4122.
164. Gritti, A., Bonfanti, L., Doetsch, F., Caille, I., Alvarez-Buylla, A., Lim, D.A., Galli, R., Verdugo, J.M., Herrera, D.G., and Vescovi, A.L. (2002) Multipotent neural stem cells reside into the rostral extension and olfactory bulb of adult rodents. *J. Neurosci.* **22**, 437–445.
165. Jacobs, B.L. (2002) Adult brain neurogenesis and depression. *Brain Behav. Immun.* **16**, 602–609.
166. Le Greves, M., Steensland, P., Le Greves, P., and Nyberg, F. (2002) Growth hormone induces age-dependent alteration in the expression of hippocampal growth hormone receptor and N-methyl-D-aspartate receptor subunits gene transcripts in male rats. *Proc. Natl. Acad. Sci. U. S. A.* **99**, 7119–7123.
167. Meller, R., Babity, J.M., and Grahame-Smith, D.G. (2002) 5-HT2A receptor activation leads to increased BDNF mRNA expression in C6 glioma cells. *Neuromolecular Med.* **1**, 197–205.
168. Monzavi, R. and Cohen, P. (2002) IGFs and IGFBPs: role in health and disease. *Baillieres Best Pract. Res. Clin. Endocrinol. Metab.* **16**, 433–447.
169. Turnley, A.M., Faux, C.H., Rietze, R.L., Coonan, J.R., and Bartlett, P.F. (2002) Suppressor of cytokine signaling 2 regulates neuronal differentiation by inhibiting growth hormone signaling. *Nat. Neurosci.* **5**, 1155–1162.
170. Zumkeller, W. (2002) IGFs and IGF-binding proteins as diagnostic markers and biological modulators in brain tumors. *Expert Rev. Mol. Diagn.* **2**, 473–477.
171. Åberg, M.A., Åberg, N.D., Palmer, T.D., Alborn, A.M., Carlsson-Skwirut, C., Bang, P., Rosengren, L.E., Olsson, T., Gage, F.H., and Eriksson, P.S. (2003) IGF-I has a direct proliferative effect in adult hippocampal progenitor cells. *Mol. Cell. Neurosci.* **24,** 23–40.
172. Åberg, N.D., Blomstrand, F., Åberg, M.A.I., Björklund, U., Carlsson, B., Carlsson-Skwirut, C., Bang, P., Rönnbäck, L., and Eriksson, P.S. (2003) Insulin-like growth factor-I increases astrocyte intercellular gap junctional communication and connexin43 expression in vitro. *J. Neurosci. Res.* **74**, 12–22.
173. Ajo, R., Cacicedo, L., Navarro, C., and Sanchez-Franco, F. (2003) Growth hormone action on proliferation and differentiation of cerebral cortical cells from fetal rat. *Endocrinology* **144**, 1086–1097.
174. Auld, D.S. and Robitaille, R. (2003) Glial cells and neurotransmission: an inclusive view of synaptic function. *Neuron* **40,** 389–400.

175. Bartke, A. (2003) Can growth hormone (GH) accelerate aging? Evidence from GH-transgenic mice. *Neuroendocrinology* **78**, 210–216.
176. Carleton, A., Petreanu, L.T., Lansford, R., Alvarez-Buylla, A., and Lledo, P.M. (2003) Becoming a new neuron in the adult olfactory bulb. *Nat. Neurosci.* **6**, 507–518.
177. Cheng, C.M., Mervis, R.F., Niu, S.L., Salem, Jr., N., Witters, L.A., Tseng, V., Reinhardt, R., and Bondy, C.A. (2003) Insulin-like growth factor 1 is essential for normal dendritic growth. *J. Neurosci. Res.* **73**, 1–9.
178. Dempsey, R.J., Sailor, K.A., Bowen, K.K., Tureyen, K., and Vemuganti, R. (2003) Stroke-induced progenitor cell proliferation in adult spontaneously hypertensive rat brain: effect of exogenous IGF-1 and GDNF. *J. Neurochem.* **87**, 586–597.
179. Doetsch, F. (2003) The glial identity of neural stem cells. *Nat. Neurosci.* **6**, 1127–1134.
180. Fukudome, Y., Tabata, T., Miyoshi, T., Haruki, S., Araishi, K., Sawada, S., and Kano, M. (2003) Insulin-like growth factor-I as a promoting factor for cerebellar Purkinje cell development. *Eur. J. Neurosci.* **17**, 2006–2016.
181. Guan, J., Bennet, L., Gluckman, P.D., and Gunn, A.J. (2003) Insulin-like growth factor-1 and post-ischemic brain injury. *Prog., Neurobiol.* **70**, 443–462.
182. Kakizawa, S., Yamada, K., Iino, M., Watanabe, M., and Kano, M. (2003) Effects of insulin-like growth factor I on climbing fibre synapse elimination during cerebellar development. *Eur. J. Neurosci.* **17**, 545–554.
183. Malberg, J.E. and Duman, R.S. (2003) Cell proliferation in adult hippocampus is decreased by inescapable stress: reversal by fluoxetine treatment. *Neuropsychopharmacology* **28**, 1562–1571.
184. Nuñez, A., Carro, E., and Torres-Aleman, I. (2003) Insulin-like growth factor I modifies electrophysiological properties of rat brain stem neurons. *J. Neurophysiol.* **89**, 3008–3017.
185. Perez-Martin, M., Azcoitia, I., Trejo, J.L., Sierra, A., and Garcia-Segura, L.M. (2003) An antagonist of estrogen receptors blocks the induction of adult neurogenesis by insulin-like growth factor-I in the dentate gyrus of adult female rat. *Eur. J. Neurosci.* **18**, 923–930.
186. Persson, A.I., Åberg, N.D., Oscarsson, J., Isaksson, O.G.P., Rönnbäck, L., Frick, F., Sonesson, C., and Eriksson, P.S. (2003) Expression of delta opioid receptor mRNA and protein in the rat cerebral cortex and cerebellum is decreased by growth hormone. *J. Neurosci. Res.* **71**, 496–503.
187. Vicario-Abejón, C., Yusta-Boyo, M.J., Fernández-Moreno, C., and de Pablo, F. (2003) Locally born olfactory bulb stem cells proliferate in response to insulin-related factors and require endogenous insulin-like growth factor-I for differentiation into neurons and glia. *J. Neurosci.* **23**, 895–906.
188. Yamada, K. and Nabeshima, T. (2003) Brain-derived neurotrophic factor/TrkB signaling in memory processes. *J. Pharmacol. Sci.* **91**, 267–270.
189. Banasr, M., Hery, M., Printemps, R., and Daszuta, A. (2004) Serotonin-induced increases in adult cell proliferation and neurogenesis are mediated through different and common 5-HT receptor subtypes in the dentate gyrus and the subventricular zone. *Neuropsychopharmacology* **29**, 450–460.
190. Bienvenu, G., Seurin, D., Grellier, P., Froment, P., Baudrimont, M., Monget, P., Le Bouc, Y., and Babajko, S. (2004) Insulin-like growth factor binding protein-6 transgenic mice: postnatal growth, brain development, and reproduction abnormalities. *Endocrinology* **145**, 2412–2420.
191. Chesik, D., De Keyser, J., and Wilczak, N. (2004) Involvement of insulin-like growth factor binding protein-2 in activated microglia as assessed in post mortem human brain. *Neurosci. Lett.* **362,** 14–16.
192. Digicaylioglu, M., Garden, G., Timberlake, S., Fletcher, L., and Lipton, S.A. (2004) Acute neuroprotective synergy of erythropoietin and insulin-like growth factor I. *Proc. Natl. Acad. Sci. U. S. A.* **101**, 9855–9860.
193. Eriksson, P.S. and Wallin, L. (2004) Functional consequences of stress-related suppression of adult hippocampal neurogenesis — a novel hypothesis on the neurobiology of burnout. *Acta Neurol. Scand.* **110**, 275–280.
194. Hsieh, J., Aimone, J.B., Kaspar, B.K., Kuwabara, T., Nakashima, K., and Gage, F.H. (2004) IGF-I instructs multipotent adult neural progenitor cells to become oligodendrocytes. *J. Cell Biol.* **164**, 111–122.
195. Huang, Y., Kim, S.O., Yang, N., Jiang, J., and Frank, S.J. (2004) Physical and functional interaction of growth hormone and insulin-like growth factor-I signaling elements. *Mol. Endocrinol.* **18**, 1471–1485.
196. Lopez-Lopez, C., LeRoith, D., and Torres-Aleman, I. (2004) Insulin-like growth factor I is required for vessel remodeling in the adult brain. *Proc. Natl. Acad. Sci. U. S. A.* **101**, 9833–9838.
197. Nagy, J.I., Dudek, F.E., and Rash, J.E. (2004) Update on connexins and gap junctions in neurons and glia in the mammalian nervous system. *Brain Res. Brain Res. Rev.* **47**, 191–215.
198. Persson, A.I., Naylor, A.S., Jonsdottir, I.H., Nyberg, F., Eriksson, P.S., and Thorlin, T. (2004) Differential regulation of hippocampal progenitor proliferation by opioid receptor antagonists in running and non-running spontaneously hypertensive rats. *Eur. J. Neurosci.* **19**, 1847–1855.
199. Porras, O.H., Loaiza, A., and Barros, L.F. (2004) Glutamate mediates acute glucose transport inhibition in hippocampal neurons. *J. Neurosci.* **24**, 9669–9673.
200. Ransome, M.I., Goldshmit, Y., Bartlett, P.F., Waters, M.J., and Turnley, A.M. (2004) Comparative analysis of CNS populations in knockout mice with altered growth hormone responsiveness. *Eur. J. Neurosci.* **19**, 2069–2079.
201. Russo, V.C., Kobayashi, K., Najdovska, S., Baker, N.L., and Werther, G.A. (2004) Neuronal protection from glucose deprivation via modulation of glucose transport and inhibition of apoptosis: a role for the insulin-like growth factor system. *Brain Res.* **1009**, 40–53.
202. Shin, D.H., Lee, E., Kim, J.W., Kwon, B.S., Jung, M.K., Jee, Y.H., Kim, J., Bae, S.R., and Chang, Y.P. (2004) Protective effect of growth hormone on neuronal apoptosis after hypoxia-ischemia in the neonatal rat brain. *Neurosci. Lett.* **354**, 64–68.

203. Verleysdonk, S., Hirschner, W., Wellard, J., Rapp, M., de los Angeles Garcia, M., Nualart, F., and Hamprecht, B. (2004) Regulation by insulin and insulin-like growth factor of 2-deoxyglucose uptake in primary ependymal cell cultures. *Neurochem. Res.* **29**, 127–134.
204. Åberg, N.D. (2005) Growth hormone and insulin-like growth factor I and their effects on astroglial gap junctions. In *The Somatotrophic Axis in Brain Function*. Nyberg, F., Ed. Elsevier, San Diego, CA. pp. 143–168.
205. Åberg, N.D., Åberg, M.A.I., and Eriksson, P.S. (2005) Growth hormone and insulin-like growth factor I and cellular regeneration in the adult brain. In *The Somatotrophic Axis in Brain Function.* Nyberg, F., Ed. Elsevier, San Diego, CA. pp. 121–141.
206. Adlard, P.A., Perreau, V.M., Pop, V., and Cotman, C.W. (2005) Voluntary exercise decreases amyloid load in a transgenic model of Alzheimer's disease. *J. Neurosci.* **25**, 4217–4221.
207. Arwert, L.I., Veltman, D.J., Deijen, J.B., Lammertsma, A.A., Jonker, C., and Drent, M.L. (2005) Memory performance and the growth hormone/insulin-like growth factor axis in elderly: a positron emission tomography study. *Neuroendocrinology* **81**, 31–40.
208. Bartke, A. (2005) Minireview: role of the growth hormone/insulin-like growth factor system in mammalian aging. *Endocrinology* **146**, 3718–3723.
209. Brywe, K.G., Mallard, C., Gustavsson, M., Hedtjärn, M., Leverin, A.-L., Wang, X., Blomgren, K., Isgaard, J., and Hagberg, H. (2005) IGF-1 neuroprotection in the immature brain after hypoxia-ischemia, involvement of Akt and GSK3b. *Eur. J. Neurosci.* **21**, 1489–1502.
210. Carro, E., Spuch, C., Trejo, J.L., Antequera, D., and Torres-Aleman, I. (2005) Choroid plexus megalin is involved in neuroprotection by serum insulin-like growth factor I. *J. Neurosci.* **25,** 10884–10893.
211. Dominici, F.P., Argentino, D.P., Muñoz, M.C., Miquet, J.G., Sotelo, A.I., and Turyn, D. (2005) Influence of the crosstalk between growth hormone and insulin signalling on the modulation of insulin sensitivity. *Growth Horm. IGF Res.* **15,** 324–336.
212. Hoshaw, B.A., Malberg, J.E., and Lucki, I. (2005) Central administration of IGF-I and BDNF leads to long-lasting antidepressant-like effects. *Brain Res.* **1037**, 204–208.
213. Le Grevès, M., Le Grevès, P., and Nyberg, F. (2005) Age-related effects of IGF-1 on the NMDA-, GH- and IGF-1-receptor mRNA transcripts in the rat hippocampus. *Brain Res. Bull.* **65**, 369–374.
214. Marshall, C.A., Novitch, B.G., and Goldman, J.E. (2005) Olig2 directs astrocyte and oligodendrocyte formation in postnatal subventricular zone cells. *J. Neurosci.* **25**, 7289–7298.
215. Nagaraja, T.N., Patel, P., Gorski, M., Gorevic, P.D., Patlak, C.S., and Fenstermacher, J.D. (2005) In normal rat, intraventricularly administered insulin-like growth factor-1 is rapidly cleared from CSF with limited distribution into brain. *Cerebrospinal Fluid Res.* **2**, 5.
216. Pan, W., Yu, Y., Cain, C.M., Nyberg, F., Couraud, P.O., and Kastin, A.J. (2005) Permeation of growth hormone across the blood-brain barrier. *Endocrinology* **146(11),** 4898–4904.
217. Perls, T.T., Reisman, N.R., and Olshansky, S.J. (2005) Provision or distribution of growth hormone for "antiaging": clinical and legal issues. *JAMA* **294**, 2086–2090.
218. Ramsey, M.M., Adams, M.M., Ariwodola, O.J., Sonntag, W.E., and Weiner, J.L. (2005) Functional characterization of des-IGF-1 action at excitatory synapses in the CA1 region of rat hippocampus. *J. Neurophysiol.* **94**, 247–254.
219. Sun, L.Y., Evans, M.S., Hsieh, J., Panici, J., and Bartke, A. (2005) Increased neurogenesis in dentate gyrus of long-lived Ames dwarf mice. *Endocrinology* **146**, 1138–1144.
220. Veiga, S., Azcoitia, I., and Garcia-Segura, L.M. (2005) Extragonadal synthesis of estradiol is protective against kainic acid excitotoxic damage to the hippocampus. *Neuroreport* **16**, 1599–1603.

### BIOSKETCHES

**N. David Åberg,** MD, PhD, is working as a clinical resident (however, the position being shared for research) at the Department of Internal Medicine, Sahlgrenska Academy, Göteborg University, Sweden. His main interest is investigating the regenerative mechanisms that the GH-IGF-I system may have in the central nervous system. He is currently focusing on preclinical studies on the effects of GHS, GH, and IGF-I regarding mechanisms of plasticity and brain repair in the rodent brain.

**Jörgen Isgaard,** MD, PhD**,** is Professor of Medicine and Consultant in Clinical Endocrinology at the Department of Internal Medicine, Sahlgrenska Academy, Göteborg University, Sweden. Dr. Isgaard has a long standing interest in cardiovascular endocrinology, predominantly basic science, but to some extent also involved in clinical studies in this area. Dr. Isgaard also has an interest in endocrine effects, in particular the GH/IGF-I axis on the CNS.

**Katarina Gustafson Brywe**, MD, PhD, is as a specialist in Obstetrics and Gynecology at Sahlgrenska University Hospital in Göteborg, Sweden. Her work as an Obstetrician and Gynecologist has focused on asphyxial brain damage caused by intrapartal events. Dr. Brywe works with an animal model with neonatal rats, inducing a hypoxic-ischemic brain damage trying to mimic the clinical situation. Furthermore she has worked with protection studies using different trophic factors as IGF-I, GH and GHS. After showing protective effects, she is trying to rule out the protective mechanisms, especially effects on apoptosis and cell survival pathways.
